# Association of IL4I1^+^ M2-like macrophages in tumor microenvironment with poor prognosis in hepatocellular carcinoma: insights from bioinformatics and experimental validation

**DOI:** 10.3389/fimmu.2026.1872272

**Published:** 2026-07-09

**Authors:** Tao Yang, Yi Lu, Yu-Qin Li, Xu-Hui Bao, Hui-Ping Lu, Wu-Jun Xiong

**Affiliations:** 1 Department of Pharmacy, Shanghai Pudong Hospital, Fudan University Pudong Medical Center, Shanghai, China; 2 Intelligent Medicine Institute, Shanghai Medical College, Fudan University, Shanghai, China; 3 Department of Gastroenterology, Shanghai Pudong Hospital, Fudan University Pudong Medical Center, Shanghai, China; 4 Institute of Therapeutic Cancer Vaccines, Shanghai Pudong Hospital, Fudan University Pudong Medical Center, Shanghai, China; 5 Center for Clinical Research, Shanghai Pudong Hospital, Fudan University Pudong Medical Center, Shanghai, China; 6 Clinical Research Center for Cell-based Immunotherapy, Fudan University, Shanghai, China; 7 Department of Pathology, Duke University Medical Center, Durham, NC, United States

**Keywords:** hepatocellular carcinoma, IL4I1, M2-like macrophages, prognosis, tumor microenvironment

## Abstract

**Background:**

Hepatocellular carcinoma (HCC) is associated with poor prognosis and limited responses to immunotherapy, partly due to the immunosuppressive tumor microenvironment (TME). Tumor-associated macrophages, especially M2-like macrophages, play important roles in HCC progression. Interleukin-4-induced gene 1 (IL4I1), a tryptophan-metabolizing enzyme, has been implicated in tumor immune regulation. However, the role and prognostic significance of IL4I1-expressing M2-like macrophages in HCC remain unclear. This study investigated the expression pattern, spatial distribution, functional association, and prognostic relevance of IL4I1-expressing M2-like macrophages in HCC and their association with patient outcomes.

**Methods:**

We analyzed public databases (TIMER, UALCAN, TISCH2) for gene expression and prognostic significance. An *in vitro* co-culture system was established using a Huh7 HCC cell line and THP-1-derived M2-like macrophages with and without IL4I1 knockout via CRISPR/Cas9. We assessed cell proliferation, migration, apoptosis, and cytokine profiles. Multiplex immunofluorescence (mIF) was performed on tissue microarrays from 92 HCC patients to analyze the spatial distribution of IL4I1+ M2-like macrophages.

**Results:**

IL4I1 was highly expressed in HCC, predominantly within macrophages, and correlated with poor prognosis. In a THP-1-derived macrophage/Huh7 co-culture model, IL4I1 expression in M2-like macrophages was associated with increased Huh7 cell proliferation and migration and reduced apoptosis. This was associated with increased secretion of pro-inflammatory cytokines (e.g., CCL15, TNF-α) and decreased levels of IL-10 and TGF-β1. mIF analysis further showed that a high density of IL4I1+ M2-like macrophages in the epithelial/parenchymal tumor region was significantly associated with poorer overall survival (*p* = 0.033).

**Conclusions:**

IL4I1+ M2-like macrophages are associated with a pro-tumorigenic microenvironment and adverse patient outcomes in HCC. Our findings support IL4I1+ M2-like macrophages as a prognostically relevant macrophage subset, while further mechanistic validation, particularly of IL4I1-derived metabolites and AHR pathway activation, is required before IL4I1 can be considered a validated therapeutic target.

## Introduction

Liver cancer remains a significant global health challenge, particularly affecting populations in East and Southeast Asia (1). Hepatocellular carcinoma (HCC), the predominant type of primary liver cancer, is the third leading cause of cancer-related deaths worldwide (2). The complex pathogenesis of HCC, coupled with its subtle early symptoms, rapid progression, and poor prognosis, has led to an increase in both incidence and mortality rates in recent years (3). Despite the availability of curative options like surgical resection and percutaneous ablation for early-stage HCC, the disease is often diagnosed at a later stage. For patients in advanced cases who are not eligible for surgical or curative procedures, palliative therapies such as tyrosine kinase inhibitors (TKIs) including sorafenib, lenvatinib, regorafenib, cabozantinib, or anti-vascular endothelial growth factor receptor 2 (VEGFR2) antibody ramucirumab are used, with sorafenib being the cornerstone of advanced HCC management over the past decade (4–6).

However, sorafenib, a front-line therapy for advanced HCC, targets the RAF-MEK-ERK pathway and inhibits angiogenesis through VEGFR2, merely offering a modest survival benefit of just 2.8 months compared to placebo (6). Since 2017, newer therapies have emerged to modulate the liver tumor microenvironment (TME), including pembrolizumab and nivolumab, which target programmed cell death-1 (PD-1), and a combination of nivolumab with ipilimumab, which targets cytotoxic T-lymphocyte-associated protein 4 (CTLA-4) (7–9). In 2020, the US FDA approved the combination of atezolizumab (anti-PD-L1) and bevacizumab (anti-VEGF) as a first-line treatment for advanced liver cancer. This regimen demonstrated superior efficacy over sorafenib, with a median progression-free survival (PFS) of 6.8 months compared to 4.3 months for sorafenib (10). Despite the promising results of immunotherapy in HCC, only a minority (20-30%) of patients derive substantial benefit from these treatments. Identifying these responders remains a significant challenge, as current biomarkers have yet to reliably predict which patients will respond favorably (11).

These findings underscore the critical need to further investigate the liver TME, a complex ecosystem comprising malignant cells, immune cells, tumor-associated macrophages (TAMs), myeloid-derived suppressive cells (MDSCs), blood vessel cells, cancer-associated fibroblasts (CAFs), non-cancerous cells, and the extracellular matrix (12). Within this intricate environment, immunosuppressive cells such as TAMs, regulatory T cells (Tregs), MDSCs, and neutrophils play pivotal roles in undermining the effectiveness of immune checkpoint inhibitors (ICIs) (13). Notably, TAMs, which occupy central immune-regulatory positions within the TME, can significantly diminish the efficacy of immune-based therapies by disrupting the communication between tumor cells and cytotoxic immune cells (14).

Interleukin-4-induced gene 1 (IL4I1), a tryptophan-metabolizing enzyme, produces metabolites such as Kynurenine (Kyn), Kynurenic Acid (KynA), and indole-3-aldehyde (I3A), which activate the Aryl Hydrocarbon Receptor (AHR). This activation not only promotes malignant features in cancer cells but also suppress T cell proliferation and function (15). Mulder et al. reported that IL4I1^+^IDO1^+^ macrophages appear to be programmed through interactions with specific T cell subsets, and IL4I1^+^ macrophages may contribute to creating an immunosuppressive TME by increasing the accumulation of regulatory T cells (16). Traditionally, macrophages were classified into two distinct activation states, M1 and M2, based on the presence of specific cell surface markers indicative of their polarization (17). However, recent studies utilizing single-cell RNA sequencing (sc-RNA-seq) and spatial transcriptomics (ST) have unveiled greater heterogeneity among TAMs in HCC. These studies identified macrophages with distinct molecular phenotypes that are closely associated with poor prognosis (18, 19). Given these findings, further characterization of IL4I1^+^ macrophages, particularly in identifying subtypes linked to poor patient outcomes and their spatial distribution, is crucial for the development of targeted immunotherapy strategies. Meanwhile, the study by Mulder et al. primarily emphasizes the interactions between macrophages and T cells (16).

Here, we used a multifaceted approach to characterize the expression pattern, cellular distribution, spatial organization, and prognostic relevance of IL4I1 in the HCC TME. First, we analyzed published datasets of sc-RNA-seq to identify the distribution of IL4I1 across different cell types within the HCC TME. Next, we employed a co-culture system of M2-like macrophages and the HCC cell line Huh7 to investigate whether IL4I1 expression in M2-like macrophages is associated with pro-tumorigenic phenotypes of HCC cells *in vitro* and to explore the accompanying changes in the cytokine microenvironment. Finally, we performed multiplex immunofluorescence analysis of clinical HCC samples to assess the spatial distribution and prognostic relevance of IL4I1+ M2-like macrophages.

## Materials and methods

### Expression analysis of IL4I1 and TDO2 in TIMER and UALCAN database

We explored IL4I1 mRNA expression across various cancer types using the Tumor Immune Estimation Resource (TIMER 2.0) (http://timer.cistrome.org/) database, a thorough resource for the systematic examination of immune infiltration across a variety of cancer types. Additionally, the UALCAN platform (http://ualcan.path.uab.edu/index.html), a tool for visualizing gene expression and survival curves, was utilized to gauge IL4I1 mRNA and protein expression levels. Moreover, we conducted an analysis examining IL4I1 expression in HCC stratified by sample type, age, sex, and tumor stage. In addition, the Tryptophan 2,3-dioxygenase (TDO2) protein expression level was also analyzed by using the UALCAN platform.

### Comparison of the gene expression across datasets at single-cell resolution

The Tumor Immune Single-cell Hub 2 (TISCH2) (http://tisch.comp-genomics.org/home/) was used to compare cell-type distribution and gene expression patterns of multiple datasets at single-cell resolution to explore the cell-type distribution and gene expression patterns of IL4I1, TDO2, Indoleamine 2,3-dioxygenase 1 (IDO1), and AHR across multiple HCC cohorts (GSE140228, GSE146409 and GSE166635).

### Analysis of prognosis and multivariate Cox regression

The prognostic significance of IL4I1 and TDO2 mRNA transcription levels was assessed utilizing KM plotter, an online open-access database (http://www.kmplot.com/) containing gene expression profiles and survival data of HCC patients. A cohort of 364 HCC patients was selected for analysis. These patients were stratified into two groups based on their gene expression levels. For each gene, patients with expression levels above the median were assigned to the high-expression group, whereas those with expression levels below the median were assigned to the low-expression group. The clinical outcome of the patients was expressed as overall survival (OS). Additionally, various statistical parameters such as hazard ratio (HR), 95% confidence intervals (CI), and log-rank P values were computed using this database. A significance threshold of *p* < 0.05 was employed to denote statistical significance.

Multivariate Cox regression analyses with a forward stepwise procedure were performed to investigate whether IL4I1 could be an independent prognostic factor for HCC patients. Clinical parameters included race, gender, age, and stages.

### Protein-protein interaction network

The STRING website (https://cn.string-db.org/) was used to assess the protein-protein interaction (PPI) network of IL4I1 and visualized by Cytoscape software.

### Immune infiltration analysis

TIMER 2.0 (https://timer.cistrome.org/) was utilized to explore the association between IL4I1 expression and tumor purity as well as the abundance of immune cells (including B cells, CD4+ T cells, CD8+ T cells, dendritic cells, macrophages, and neutrophils). We also investigated the relationship between clinical prognosis of HCC patients and B cells, CD4+ T cells, CD8+ T cells, dendritic cells, macrophages, and neutrophils with TIMER 2.0. Based on the quartiles of gene expression, all patients were categorized into four types, i.e., Q1, Q2, Q3 and Q4, with Q1 representing the 25% of samples with the highest expression of a particular gene and Q4 representing the 25% of samples with the lowest expression. Following the previous study by Thorsson V et al. on immune response and genomic status (doi: 10.1016/j.immuni.2019.08.004.), the mean value of each scoring across the four patient types was calculated and visualized using the “pheatmap” package. Matrix files of microbial abundance were downloaded from the TCMA database and Spearman correlations between microbial abundance and patient gene expression were calculated.

### Evaluation of immune cell composition

Based on the HCC expression data from TCGA, we performed immune cell composition and comparative analysis using the CIBERSORT R package. First, we prepared three files - the CIBERSORT gene signature file LM22, the TCGA gene expression matrix, and the CIBERSORT code. Then, based on the analysis results, we created histograms and box plots to display the proportions of various immune cell compositions within the samples. Additionally, we generated comparative box plots to illustrate the differences in immune cell compositions between the samples with high IL4I1 expression and those with low expression.

### Correlation analysis between IL4I1 and macrophage-associated marker genes

To further clarify the relationship between IL4I1 expression and macrophage-associated transcriptional programs, we performed an additional marker-gene correlation analysis using TCGA-LIHC RNA-seq data. Gene expression data from primary tumor samples were obtained from the TCGA-LIHC cohort and normalized as transcripts per million (TPM). Expression values were log2-transformed as log2(TPM + 1) before analysis. Representative macrophage, M2-like, and TAM-associated marker genes were selected, including CD68, CSF1R, LST1, CD163, MRC1, MSR1, MS4A4A, and VSIG4. Additional immune activation-related markers, including CD86, IL1B, and CXCL9, were also included to explore the broader macrophage activation context. Spearman correlation analysis was used to assess the association between IL4I1 and each marker gene. P values were adjusted for multiple testing using the Benjamini–Hochberg method, and an adjusted p value < 0.05 was considered statistically significant. The analysis was performed using R software.

### Generation of IL4I1 knockout in THP-1 monoclonal cells and polarization

Human IL4I1 gene (Gene ID: 259307) was knocked out in human monocytic leukemia cell line THP-1 cells using Clustered Regularly Interspaced Short Palindromic Repeats/Cas9 (CRISPR/Cas9) technology. A lentiviral vector carrying sgRNA (single guide RNA) (5’-GGTAGATGTCTTCGGGCGAGTGG-3’) targeting IL4I1 was constructed and packaged in human embryonic kidney 293T cell line (HEK293T) cells. THP-1 cells were infected and selected with puromycin before single-cell cloning. Genome editing was confirmed by sequencing. For functional validation, THP-1 cells with IL4I1 knockout (THP-1-IL4I1-KO) and control THP-1 cells with negative control sgRNA (THP-1-sgNC) were differentiated using phorbol 12-myristate 13-acetate (PMA, 50 ng/mL, 24 h) and polarized to the M2 phenotype with Interleukin-4 (IL-4, 20 ng/mL, 48 h). This process generated KO-M2-like macrophages (referred to as KO-M2) and NC-M2-like macrophages (referred to as NC-M2), respectively. IL4I1 mRNA expression was quantified by RT-qPCR using specific primers, with Glyceraldehyde-3-phosphate dehydrogenase (GAPDH) as reference gene.

### Flow cytometric validation of M2-like macrophage polarization

Following polarization, the KO-M2 and NC-M2-like macrophages were harvested and resuspended in 1× Perm/Wash buffer, followed by centrifugation. The cells were then incubated with CD206 and CD11b antibodies in a staining buffer and kept in the dark for 30 minutes. After washing twice with 1× Perm/Wash buffer, cells were resuspended in staining buffer and analyzed using a BD FACSVerse flow cytometer. The expression levels of M2 markers (CD11b^+^CD206^+^) were compared between KO-M2 and NC-M2 groups.

### Co-culture with Huh7 cells and functional assays

In the cell proliferation assay, Huh7 cells were cultured in DMEM medium supplemented with 10% FBS and penicillin-streptomycin. Huh7 cells were co-cultured with either KO-M2 or NC-M2 cells in 96-well plates at a density of 3 × 10^4^ cells/mL for 72 hours. Cell proliferation was assessed using the CCK-8 assay. After 72 hours of co-culture, 10 μL of CCK-8 solution was added to each well and incubated for 2 hours. The optical density (OD) was measured at 450 nm using a microplate reader.

In the cell apoptosis assay, Huh7 cells were co-cultured with KO-M2 or NC-M2 cells as described above. Apoptosis was evaluated using the Annexin V-FITC/PI Apoptosis Detection Kit. Cells were collected, washed with PBS, and resuspended in a binding buffer. Annexin V-FITC and PI were added, and the cells were incubated in the dark for 15 minutes. Apoptosis was analyzed using a BD FACSVerse flow cytometer.

In cell migration assay, transwell assays were performed to assess cell migration. Huh7 cells were seeded in the upper chamber of Transwell plates at a density of 3 × 10^5^ cells/mL. The lower chamber contained a culture medium with either KO-M2 or NC-M2 cells. After 72 hours of incubation at 37 °C with 5% CO2, non-migrated cells in the upper chamber were removed, and migrated cells were fixed with 4% paraformaldehyde and stained with crystal violet. Migrated cells were counted under a microscope.

In the wound healing assay, Huh7 cells were seeded in 3.5 cm dishes and cultured until 90% confluency. A uniform scratch was made using a 200 μL pipette tip, and the cells were washed with PBS to remove debris. Fresh medium containing either KO-M2 or NC-M2 cells was added. The wound area was photographed at 0, 24, and 48 hours using an inverted microscope to assess cell migration.

All experiments were performed in triplicate. Data were analyzed using GraphPad Prism software and presented as mean ± SD. Statistical significance was determined by one-way ANOVA and *post hoc* Tukey’s test. *p* < 0.05 were considered statistically significant.

### Tissue microarray and mIF

Commercially acquired tumor tissue microarrays (TMA), designated HlivH180su19, were obtained from Shanghai Biotech Company. These TMAs comprised 92 paired samples, each containing both HCC tissue and its corresponding adjacent normal liver tissue. Clinical follow-up data, spanning a range of 0 to 120 months, was also included. A previously described method was employed for the immunofluorescence staining (20). For the assay, we employed the Opal 7-colour Manual IHC Kit (NEL801001KT) from PerkinElmer and VECTASHIELD^®^ HardSet Antifade Mounting Medium (H-1400) from Vector Labs.

Initially, tissue sections were baked at 63 °C for 1 hour to remove paraffin, followed by deparaffinization with a fully automatic dyeing machine (LEICAST5020, LEICA) using a sequence of xylene and ethanol solutions of varying concentrations. Antigen retrieval was performed by diluting a 10x retrieval solution to 1x working solution, boiling it in a microwave on high for 3 minutes, and then continuing at low power for 15–20 minutes, after which the slides were cooled to room temperature and rinsed with distilled water. To block endogenous peroxidase activity, slides were incubated in an H_2_O_2_ solution for 10 minutes in a humidified chamber and then washed with Tris-Buffered Saline with Tween-20 (TBST) buffer. A blocking buffer was applied to the slides, which were incubated at room temperature for 10 minutes to block nonspecific binding. Following blocking, the slides were incubated with diluted primary antibodies at room temperature for 1 hour and then washed with TBST buffer. Secondary antibodies were applied, and the slides were incubated for 10 minutes at room temperature, followed by another TBST buffer wash. Opal dye diluted 1:100 was used for staining, with slides incubated for 10 minutes at room temperature and then washed with TBST buffer. This staining process was repeated for each marker until all markers were labeled. Finally, the slides were incubated with 4’,6-diamidino-2-phenylindole (DAPI) solution for 5 minutes to stain the nuclei, washed with TBST buffer, and mounted with an antifade mounting medium.

### Fluorescence signal quantification

We conducted panoramic multispectral scanning of slides using the TissueFAXS Spectra system (TissueGnostics). The resulting data was then imported into Strata-Quest analysis software. Spectral splitting, utilizing a spectral library, allowed us to obtain single-channel fluorescence signals. Nuclei were identified using the DAPI channel. For each protein channel, we established a distance radius around the nucleus to locate protein fluorescent staining signals. Thresholds were set for each channel to define positive cell populations and facilitate cell counting. We also quantified double-positive cells, both in number and intensity. To calculate the mean of gene expression, we multiplied the mean intensity by the number of positive cells and divided by the total cell count.

### ELISA

The concentrations of 12 cytokines, including Chemokine (C-C motif) ligand 2 (CCL2), Chemokine (C-C motif) ligand 15 (CCL15), Chemokine (C-C motif) ligand 17 (CCL17), Chemokine (C-C motif) ligand 20 (CCL20), Chemokine (C-X-C motif) ligand 2 (CXCL2), Chemokine (C-X-C motif) ligand 12 (CXCL12), Interleukin-1 beta (IL-1β), Interleukin-6 (IL-6), Interleukin-8 (IL-8), Interleukin-10 (IL-10), Tumor necrosis factor alpha (TNF-α), and Transforming growth factor beta 1(TGF-β1), in the supernatants collected after 72 hours of culture from Huh7 monoculture, Huh7 co-cultured with IL4I1-knockout M2-like macrophages (KO-M2), and Huh7 co-cultured with control M2-like macrophages (NC-M2), were quantified. Cytokine levels were measured using enzyme-linked immunosorbent assay (ELISA) kits (Sumeike Biotechnology, Jiangsu, China), and absorbance was recorded at 450 nm using a microplate reader (Multiskan MS, Labsystems, Finland).

### Statistical analysis

All statistical analyses were performed using GraphPad Prism software (Version 9.0.0, GraphPad Software, LLC) and R software (Version 4.3.0). Data from in vitro experiments, which were performed in triplicate, are presented as the mean ± standard deviation (SD). Comparisons between groups were performed using a one-way analysis of variance (ANOVA) followed by Tukey’s post hoc test. Survival curves were generated using the Kaplan-Meier method and compared using the log-rank test. Multivariate analysis of factors influencing overall survival was conducted using the Cox proportional hazards regression model. Correlations between gene expression and immune infiltration or other variables were assessed using Spearman’s or Pearson’s correlation coefficients where appropriate. A p-value of less than 0.05 was considered statistically significant.

## Results

### IL4I1 is upregulated at the mRNA and protein levels in HCC

**Figure 1:**
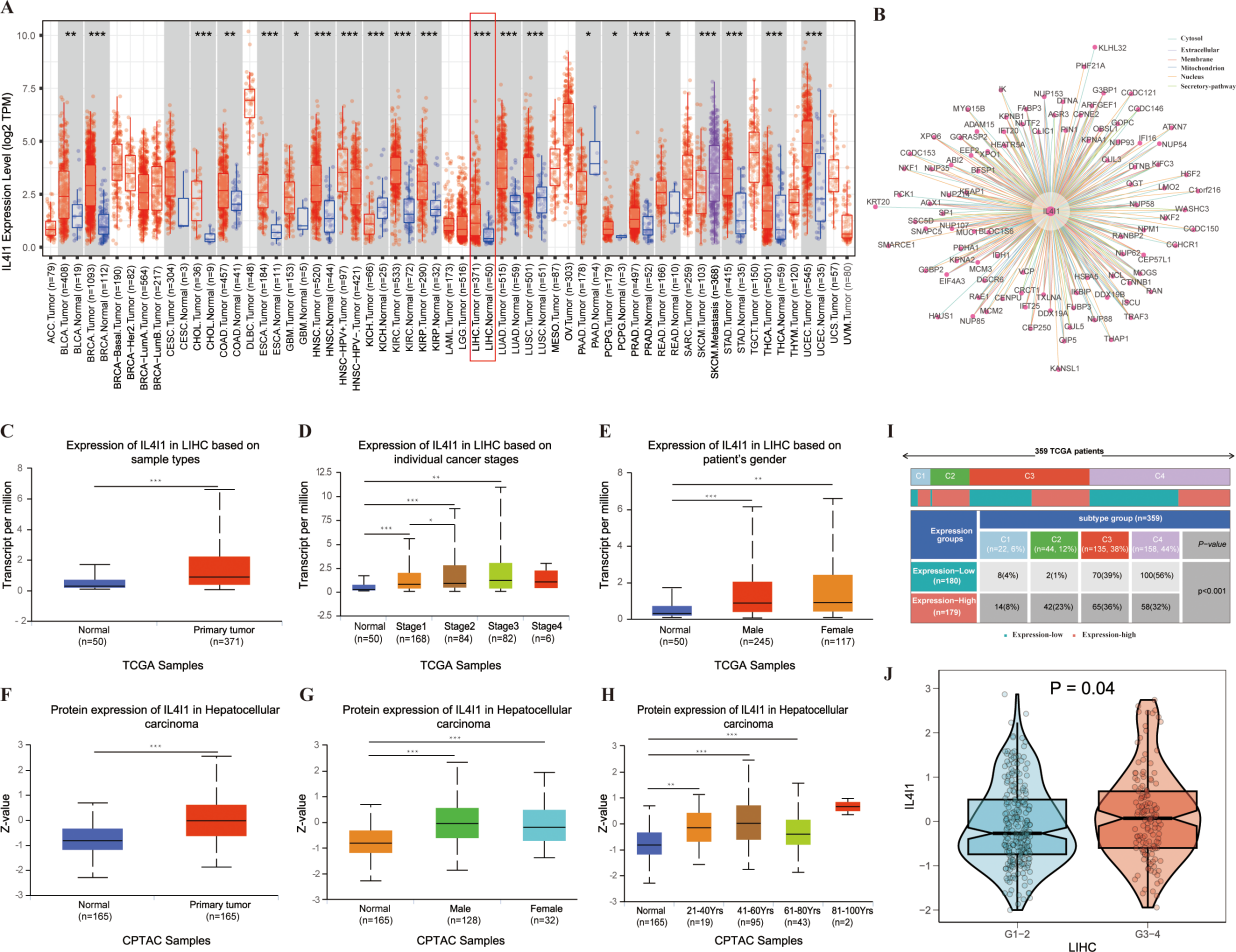
The IL4I1 expression levels in HCC. **(A)** TIMER2.0 was used to analyze IL4I1 expression levels in various tumor types from the TCGA database, with the expression in HCC highlighted in the red box; **(B)** Protein-protein interaction network of IL4I1. **(C-H)** The mRNA and protein expression level of IL4I1 in HCC based on sample type, stage, gender, and age by UALCAN database. **(I)** In pancancer, four immune subtypes are different in high and low IL4I1 expression groups. **(J)** Expression differences of genes in grades1–2 and grades 3–4 in the TCGA-LIHC cohort. ^*^p < 0.05, ^**^p < 0.01, ^***^p < 0.001.

We utilized the TIMER database to identify differences in IL4I1 mRNA expression between various tumor and normal samples. The expression levels of IL4I1 were upregulated in HCC (**Figure 1A**). PPI network suggests strong interactions between IL4I1 and many intra- and extracellular proteins as well as membrane proteins (**Figure 1B**). By analyzing data from the UALCAN database, we found that IL4I1 expression was markedly higher in HCC tumors compared to normal samples at both the mRNA and protein levels (*p* < 0.001) (**Figures 1C, F**).

We further conducted a comprehensive analysis of IL4I1 expression levels in HCC, considering various clinical factors, including stages, gender, and age. Our findings revealed that both mRNA and protein levels of IL4I1 were significantly elevated in males and females compared to normal samples (**Figures 1E, G**) (mRNA level: *p* < 0.001; protein level: *p* < 0.001, respectively). Moreover, the results showed that mRNA levels of IL4I1 were significantly higher in stage I-III but IV than in normal samples (**Figure 1D**).

In addition, we observed that IL4I1 protein expression levels were significantly elevated in individuals aged 21 to 40 years, 41 to 60 years, and 61 to 80 years except for 81 to 100 years compared to normal samples (**Figure 1H**). Analysis based on TCGA-LIHC data revealed the distribution of immune subtypes within IL4I1 high and low expression groups (**Figure 1I**). In the high IL4I1 expression group, C3 (Inflammatory) and C4 (Lymphocyte depleted) were the most common immune subtypes observed, with C3 being slightly more frequent. Notably, the C4 subtype was found to be the most predominant immune subtype within the low IL4I1 expression group (**Figure 1I**). Analysis based on TCGA-LIHC data shows that the higher the tumor stage, the higher the expression level of the IL4I1 gene (**Figure 1J**).

### Single-cell analysis identifies macrophages as a major IL4I1-expressing cell population in HCC

**Figure 2:**
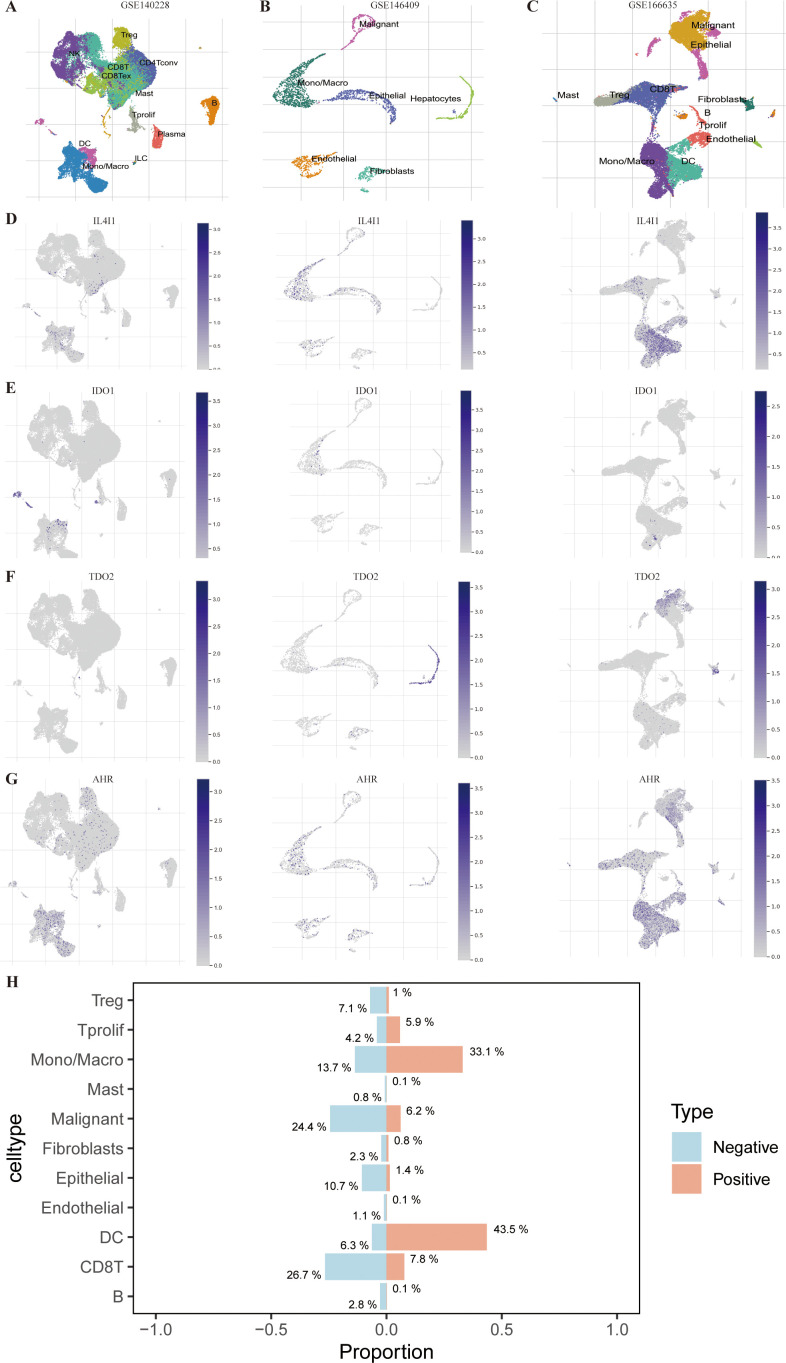
The expression profile of IL4I1, IDO1, TDO2, and AHR in different subtypes of HCC cells from different HCC samples. **(A)** UMAP plots displayed GSE140228, **(B)** GSE146409, and **(C)** GSE166635 according to the TISCH2 database. **(D)** UMAP plots show the expression of IL4I1, IDO1 **(E)**, TDO2 **(F)** and AHR **(G)** in different datasets. **(H)** Percentage of IL4I1 positive and negative cells in the GSE166635 dataset.

Using TISCH2, we analyzed the cell-type distribution and gene expression patterns in HCC datasets GSE140228, GSE146409 and GSE166635 from Gene Expression Omnibus (GEO). The most consistent findings across all three datasets indicate that IL4I1 exhibited predominantly high expression levels within monocytes/macrophages (**Figures 2A–D**). IDO1, too, was primarily expressed within monocytes/macrophages but appeared to be particularly concentrated in a specific subtype (**Figures 2A–C, E**). In contrast, TDO2 was mainly expressed in malignant liver tumor cells, with virtually no expression observed in immune cells (**Figures 2A–C, F**). AHR, on the other hand, exhibited substantial expression in both immune cells and malignant liver tumor cells (**Figures 2A–C, G**). The percentage analysis showed a higher proportion of IL4I1-positive cells among monocytes/macrophages and dendritic cells (**Figure 2H**).

### High IL4I1 expression is associated with poorer overall survival in HCC

**Figure 3:**
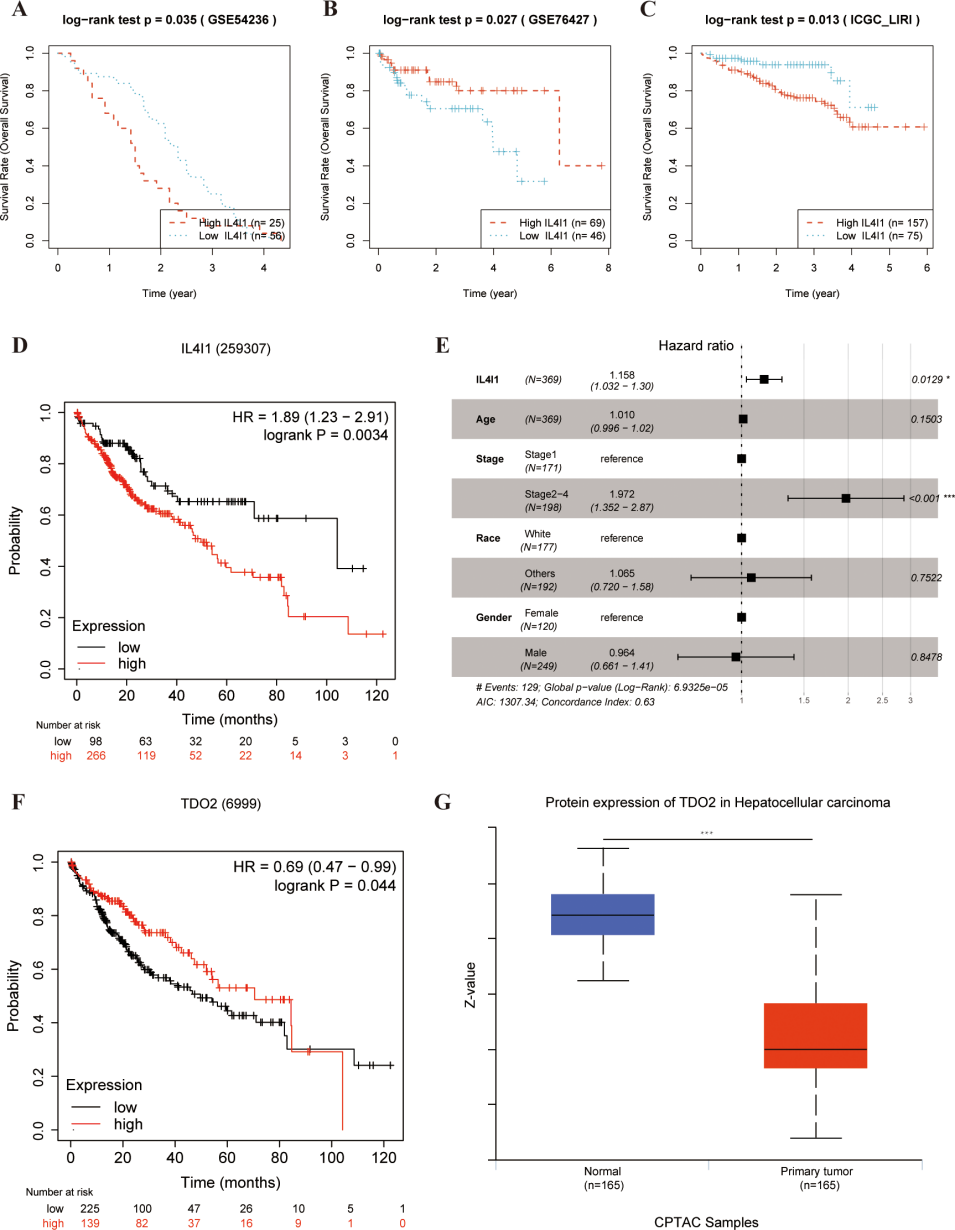
The analysis of the KM plotter, multivariate Cox regression, and protein expression of IL4I1 and TDO2. **(A-D, F)** KM plotter analysis is used to predict the correlation between IL4I1 and TDO2 expression and the overall survival of HCC patients. **(E)** Multivariate analysis of factors influencing overall survival using the Cox regression model. **(G)** The protein expression level of TDO2 in HCC based on sample type.

Using the KM plotter, which accesses public microarray expression data for survival analyses, we examined the effect of IL4I1 and TDO2 expression on OS of HCC patients. In addition to the GSE76427 dataset, high IL4I1 expression was associated with shorter OS in HCC patients (**Figures 3A–D**) (*p *< 0.05); in contrast, high expression of TDO2 predicted a longer OS (**Figure 3F**) (*p *< 0.05), meanwhile the protein expression level of TDO2 in HCC was significantly downregulated compared to normal samples (**Figure 3G**).

In patients with HCC, elevated IL4I1 expression was independently associated with unfavorable prognosis (**Figure 3E**). Simultaneously, we explored whether patient gender, age, tumor stages and race played a role as independent prognostic factors in HCC. Our findings revealed that aside from high IL4I1 expression, advanced tumor stage was also identified as an independent risk factor associated with poorer patient prognosis.

### IL4I1 expression is associated with immune-cell infiltration, and macrophage infiltration is linked to poorer prognosis

**Figure 4:**
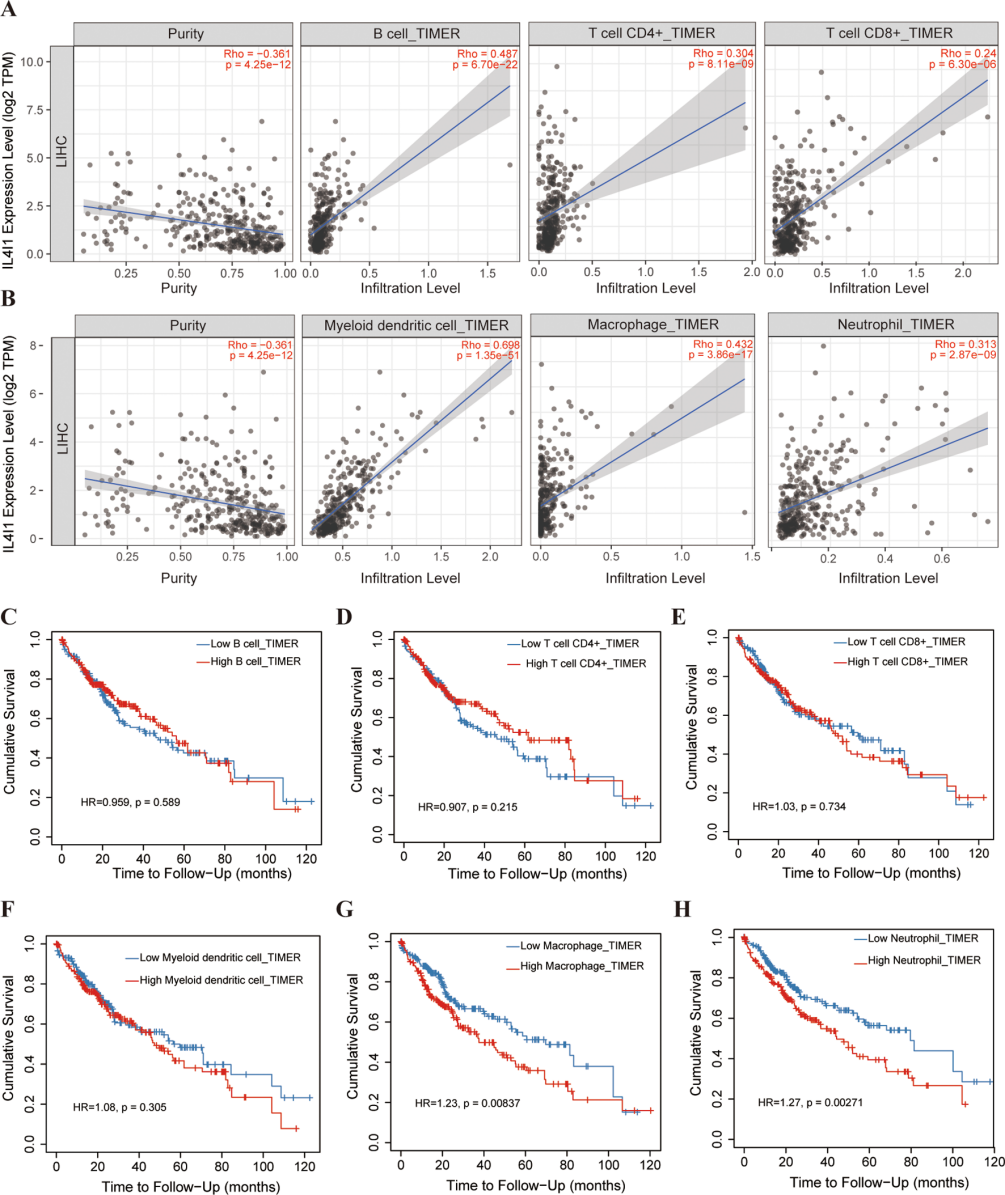
The correlations between IL4I1 expression level and infiltration level of different immune cells and the effect of infiltration level on HCC patient survival. **(A)** The correlation between IL4I1 expression level and infiltration level of B cell, CD4^+^ T cell, and CD8^+^ T cell and **(B)** Myeloid dendritic cell, Macrophage, and Neutrophil. **(C-H)** The effect of infiltration level of B cell, CD4^+^ T cell, CD8^+^ T cell, Myeloid dendritic cell, Macrophage, and Neutrophil on HCC patient survival.

TIMER2.0 was employed to evaluate the connection between IL4I1 expression and immune cell infiltration in patients with HCC. The findings indicated significant associations between IL4I1 expression and the infiltration levels of various immune cell types, including B cell (Rho = 0.487, *p* = 0.00791), CD4^+^ T cells (Rho = 0.304, *p* < 0.001), CD8^+^ T cells (Rho = 0.24, *p* < 0.001), Myeloid dendritic cell (Rho = 0.698, *p* < 0.001), Macrophage (Rho = 0.432, *p* < 0.001), Neutrophil (Rho = 0.313, *p* < 0.001). Meanwhile, the effect of infiltration level of B cell, CD4^+^ T cell, CD8^+^ T cell, Myeloid dendritic cell, Macrophage, and Neutrophil on HCC patient survival was also evaluated in TIMER2.0 (**Figures 4A, B**). High levels of macrophage and neutrophil infiltration were significantly associated with poor prognosis in HCC patients (HR = 1.23, *p* = 0.00837; HR = 1.27, *p* = 0.00271) (**Figures 4C–H**).

### Bulk transcriptomic deconvolution reveals a complex immune landscape associated with IL4I1 expression

**Figure 5:**
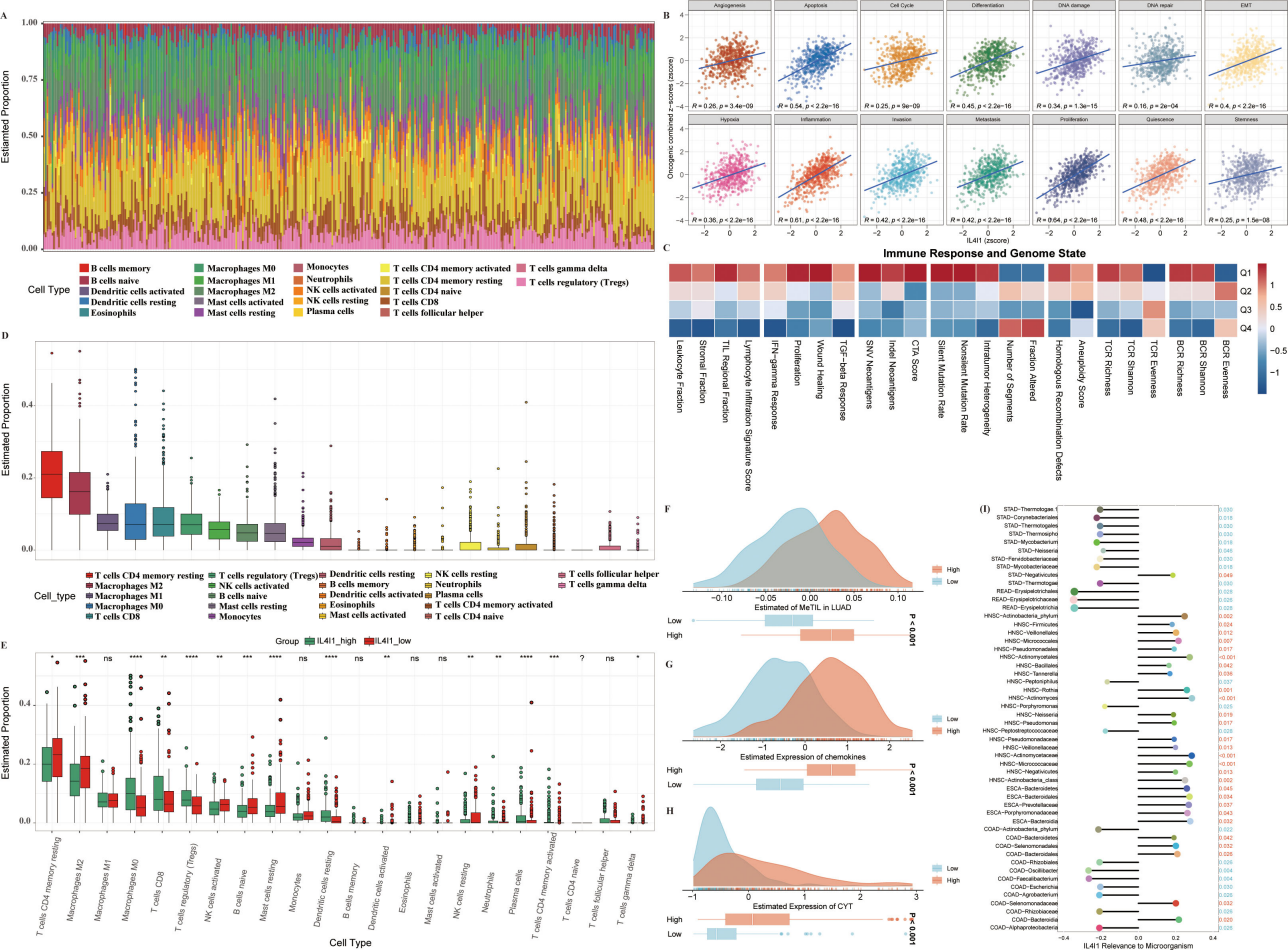
Relationship between IL4I1 expression and immune cells in HCC. **(A, D)** Relative proportions of 22 subtypes of tumor-infiltrating immune cells for each sample in HCC from TCGA by CIBERSORT. **(B)** Pearson correlation of GSVA scores between zscores of IL4I1 expression level and 14 tumor states. **(C)** Differences in expression of immunostimulatory genes, immunosuppressive genes, chemokines and human leukocyte antigen in Q1, Q2, Q3 and Q4 IL4I1 expression groups. **(E)** Comparison of the immune cell fraction difference between the low and high IL4I1 expression groups. **(F)** Differences in the expression of MeTIL, chemokines **(G)** and CYT **(H)** between high and low IL4I1 expression groups. **(I)** Spearman’s correlation between intratumoral microbial content and IL4I1 expression in the TCMA database.

**Figure 6:**
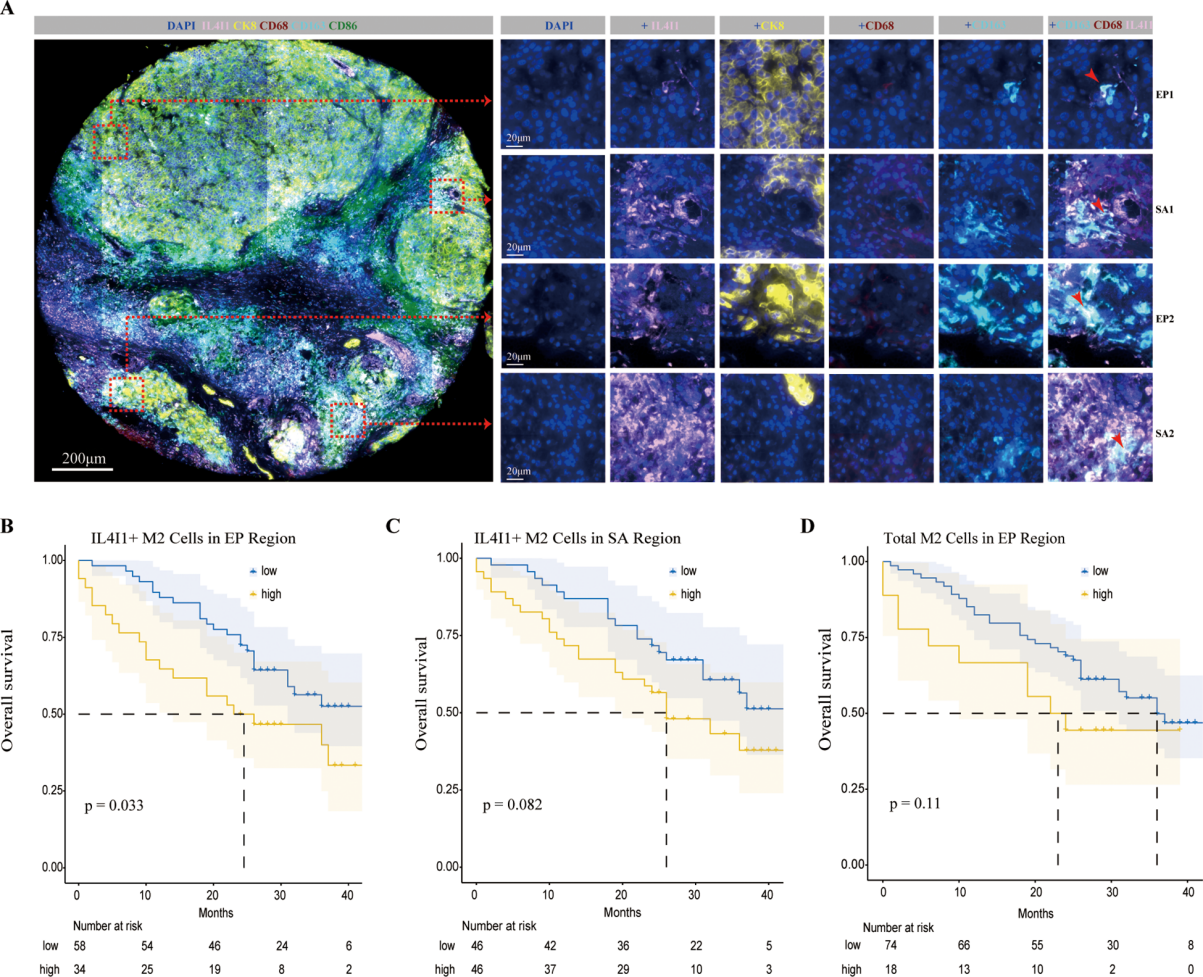
Spatial distribution and prognostic relevance of IL4I1+ M2-like macrophages in HCC tissues. **(A)** Representative multiplex immunofluorescence image showing the spatial distribution of IL4I1, CK8, CD68, CD163, CD86, and DAPI in an HCC tissue section. Red dashed boxes and connecting arrows indicate selected epithelial/parenchymal tumor regions (EP1 and EP2) and stromal areas (SA1 and SA2) shown at higher magnification. The right panels display individual and merged marker channels in the selected regions. Red arrowheads in the merged panels indicate representative IL4I1+CD68+CD163+ M2-like macrophages located in close spatial proximity to CK8+ tumor-cell areas. Scale bars: 200 μm in the panoramic image and 20 μm in the high-magnification panels. **(B)** Kaplan–Meier survival curves comparing overall survival between patients with high and low numbers of IL4I1+ M2-like macrophages in the epithelial/parenchymal tumor region. **(C)** Kaplan–Meier survival curves comparing overall survival between patients with high and low numbers of IL4I1+ M2-like macrophages in the stromal area. **(D)** Kaplan–Meier survival curves comparing overall survival between patients with high and low total numbers of M2-like macrophages in the epithelial/parenchymal tumor region. EP, epithelial/parenchymal tumor region; SA, stromal area; HCC, hepatocellular carcinoma; mIF, multiplex immunofluorescence.

To assess the bulk tumor immune landscape in relation to IL4I1 expression, we applied CIBERSORT analysis to the TCGA-LIHC cohort (n=369). This confirmed macrophages and T cells as prominent immune populations in HCC (**Figures 5A, D**). Comparing IL4I1 high versus low expression groups (**Figure 5E**), higher IL4I1 expression associated with increased estimated proportions of M0 macrophages, CD8+ T cells, and Tregs. Conversely, CIBERSORT estimated lower M2-like macrophages proportions in the IL4I1-high group. This latter finding, based on bulk RNA-seq deconvolution, differs from our direct mIF quantification (**Figure 6**) showing high density of the specific IL4I1+ M2 subset correlating with poor prognosis, highlighting the distinction between bulk estimates and specific subset analysis.

Consistent with increased immune activity signatures in IL4I1-high tumors, we observed positive correlations between IL4I1 expression and various immune/tumor states (**Figure 5B**), immune pathway activation (**Figure 5C**), and higher scores for Melanoma-associated T cell Infiltration score (MeTIL), chemokines, and cytolytic activity score (CYT) (**Figures 5F–H**). Analysis of intratumoral microbial content showed varied correlations across cancer types (**Figure 5I**).

### IL4I1 expression is associated with macrophage/TAM-related transcriptional markers in TCGA-LIHC

Because CIBERSORT estimated a lower relative proportion of M2-like macrophages in the IL4I1-high group, whereas mIF identified a prognostically relevant IL4I1+CD68+CD163+ M2-like macrophage subset, we further examined the relationship between IL4I1 and macrophage-associated marker genes in TCGA-LIHC. Spearman correlation analysis showed that IL4I1 expression was positively correlated with multiple macrophage/TAM-associated markers, including CD68 (rho = 0.358, FDR-adjusted *p* = 1.30E-12), CSF1R (rho = 0.689, FDR-adjusted *p* = 6.49E-53), LST1 (rho = 0.707, FDR-adjusted *p* = 8.19E-57), MSR1 (rho = 0.628, FDR-adjusted *p* = 9.92E-42), MS4A4A (rho = 0.557, FDR-adjusted *p* = 2.19E-31), and VSIG4 (rho = 0.531, FDR-adjusted *p* = 3.37E-28). IL4I1 was also positively correlated with the M2/TAM-related marker CD163 (rho = 0.481, FDR-adjusted *p* = 7.82E-23), while its correlation with MRC1 was positive but weak (rho = 0.135, FDR-adjusted *p* = 0.009). These findings suggest that IL4I1 expression is associated with macrophage/TAM-related transcriptional programs, although it is not equivalent to the total CIBERSORT-estimated M2-like macrophages fraction. Detailed results are shown in **Supplementary Figure 6**.

### CRISPR/Cas9-mediated IL4I1 knockout is validated in THP-1 cells

CRISPR/Cas9-mediated knockout of IL4I1 in THP-1 cells was successfully achieved, with sequence analysis of the KO#3 clone revealing a 13 bp deletion and 1 bp insertion that caused a frameshift mutation. RT-qPCR confirmed significantly reduced IL4I1 expression in THP-1-IL4I1-KO cells compared to both parental THP-1 and THP-1-sgNC control cells. NC-M2-like macrophages, generated by polarizing THP-1-sgNC cells with IL-4, showed significantly upregulated IL4I1 expression compared to their unpolarized counterparts. While IL4I1 expression remained substantially lower in polarized KO-M2 cells than in NC-M2 cells, a slight but significant increase was observed in KO-M2 cells compared to their unpolarized counterparts, suggesting possible compensatory mechanisms during M2 polarization (**Supplementary Figures 1, 2**).

### IL4I1 expression in THP-1-derived M2-like macrophages is associated with enhanced Huh7 proliferation and migration *in vitro*

**Figure 7:**
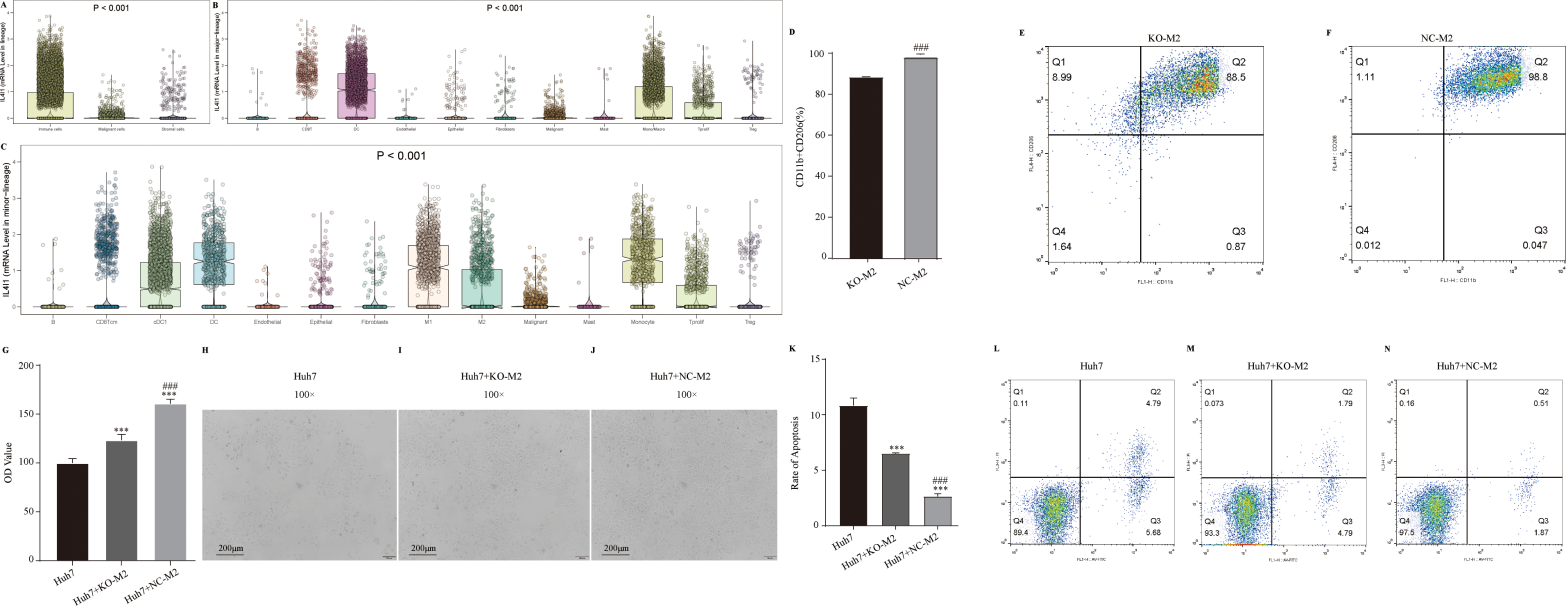
IL4I1 expression in THP-1-derived M2-like macrophages is associated with enhanced Huh7 proliferation and reduced apoptosis *in vitro*. **(A-C)** Differential violin plot of IL4I1 expression in single cell analysis. **(D-F)** Flow cytometry analysis of CD11b and CD206 expression in KO-M2 and NC-M2-like macrophages. **(G)** CCK8 assay results show the proliferation of Huh7 cells co-cultured with KO-M2 or NC-M2-like macrophages. Data are presented as mean OD values ± SD. **(H-J)** Huh7 cells co-cultured with KO-M2-like macrophages, and Huh7 cells co-cultured with NC-M2-like macrophages after 72 hours of incubation. **(K-N)** Flow cytometry analysis of apoptosis in Huh7 cells co-cultured with KO-M2 or NC-M2-like macrophages. The bar graph represents the rate of apoptosis. ^***^P < 0.001 compared to Huh7 control group; ^###^*P* < 0.001 compared to Huh7+KO-M2.

Firstly, single-cell analyses revealed that IL4I1 was predominantly expressed in M1 macrophages, monocytes and DC cells (**Figures 7A–C**). Flow cytometry data showed that the expression of CD11b^+^CD206^+^ (M2 polarization marker) was significantly upregulated in the NC-M2 polarization group compared to the KO-M2 polarization group (**Figures 7D–F**).

The CCK8 assay results demonstrated that co-culture with KO-M2 polarized macrophages significantly promoted the proliferation of Huh7 cells compared to the Huh7 control group. The NC-M2 polarized macrophages further enhanced cell proliferation (**Figures 7G–J**). In the cell apoptosis assay, flow cytometry analysis indicated a decrease in apoptosis rates in Huh7 cells co-cultured with KO-M2-like macrophages compared to the Huh7 control group. The NC-M2-like macrophages further reduced the apoptosis rates (**Figures 7K–N**).

The mIF analysis revealed the spatial distribution of IL4I1 and its co-localization with various cell lineage markers (**Supplementary Figures 3A–F**). IL4I1 protein expression (visualized in pink) was detected in multiple cell types within the tissue sections, as indicated by DAPI counterstaining (blue) showing widespread cell presence (**Supplementary Figure 3A**). Importantly, while previous RNA analyses indicated predominant IL4I1 expression in myeloid cells (**Figure 1**, **2**), our *in situ* protein analysis using mIF demonstrated detectable IL4I1 protein in other cell types as well. Specifically, co-staining with the tumor cell marker Cytokeratin 8 (CK8, yellow) revealed co-localization, indicating the presence of IL4I1 protein within a subset of tumor cells (**Supplementary Figure 3B**). Furthermore, IL4I1 protein was clearly detected in CD68+ macrophages (red) (**Supplementary Figure 3C**). Co-staining with additional markers allowed for identification within specific macrophage subsets: IL4I1 expression was observed in CD68+CD86+ M1-like macrophages (CD86 in green) (**Supplementary Figure 3D**) and also in CD68+CD163+ M2-like macrophages (CD163 in cyan) (**Supplementary Figure 3E**). The merged image (**Supplementary Figure 3F**) illustrates the complex spatial interplay between IL4I1-expressing cells (including both macrophages and tumor cells) and different cellular components within the HCC microenvironment.

### IL4I1 expression in M2-like macrophages is associated with enhanced Huh7 migration *in vitro*

**Figure 8:**
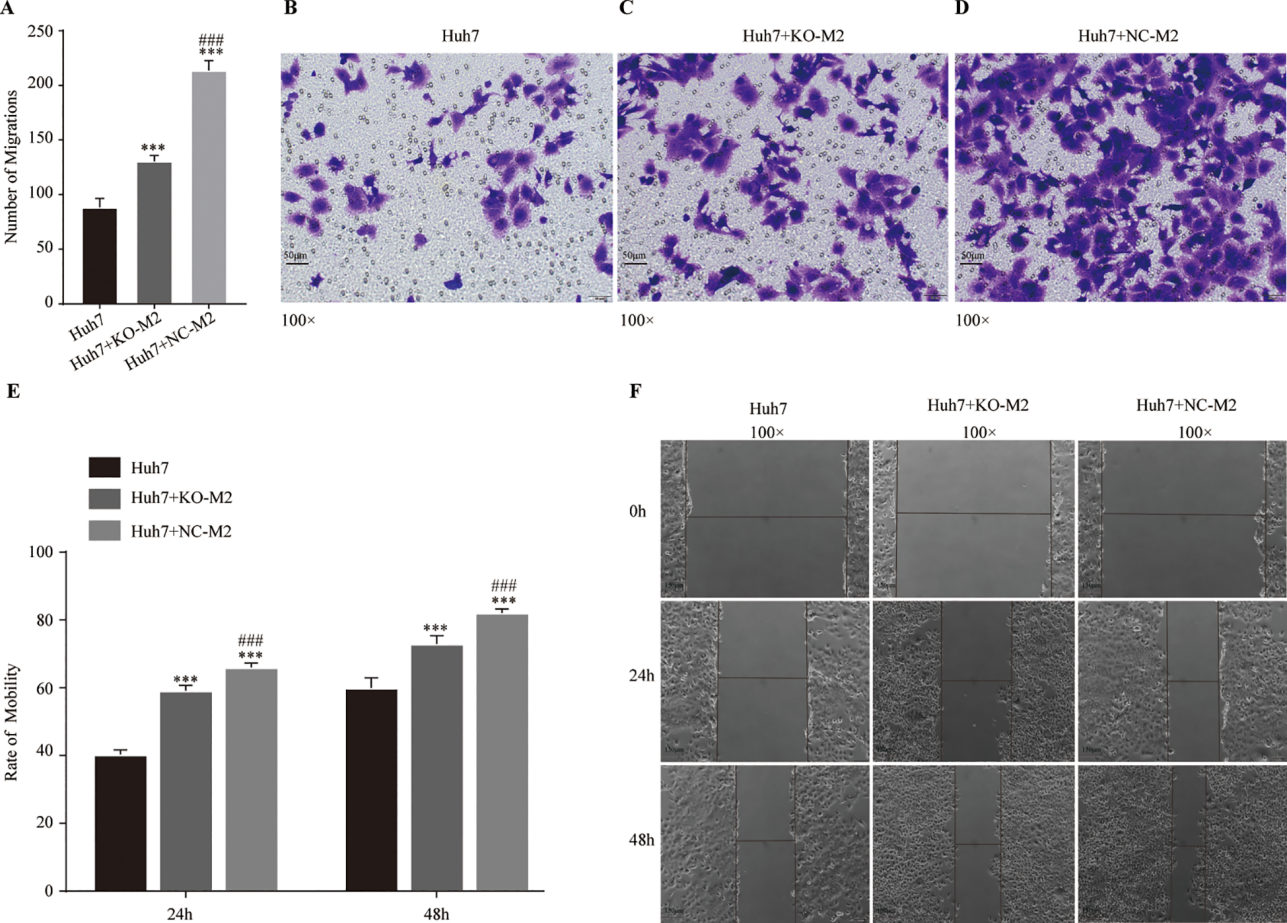
The IL4I1 effects on Huh7 cell migration and mobility. **(A)** Quantification of cell migration using the Transwell assay. The bar graph represents the number of migrated Huh7 cells co-cultured with KO-M2 or NC-M2-like macrophages. **(B-D)** Huh7 cells alone, Huh7 cells co-cultured with KO-M2-like macrophages, and Huh7 cells co-cultured with NC-M2-like macrophages. **(E)** Wound healing assay results show the rate of mobility of Huh7 cells co-cultured with KO-M2 or NC-M2-like macrophages at 24h and 48h. **(F)** Representative images of the wound healing assay at 0h, 24h, and 48h. (left) Huh7 cells alone, (center) Huh7 cells co-cultured with KO-M2-like macrophages, and (right) Huh7 cells co-cultured with NC-M2-like macrophages.

Transwell migration assay results revealed that the migration rate of Huh7 cells increased significantly when co-cultured with KO-M2-like macrophages compared to the Huh7 control group. The NC-M2-like macrophages led to an even greater increase in migration rates (**Figures 8A–D**). The wound healing assay demonstrated enhanced migration capability in Huh7 cells co-cultured with KO-M2-like macrophages at both 24h and 48h compared to the Huh7 control group. The NC-M2-like macrophages exhibited a further increase in migration capability (**Figures 8E, F**).

### Spatial localization of IL4I1+ M2-like macrophages and their association with poorer survival in HCC

Representative mIF analysis showed that IL4I1+CD68+CD163+ M2-like macrophages were detectable in both epithelial/parenchymal tumor regions and stromal areas of HCC tissues (**Figure 6A**). In selected high-magnification fields, these cells were observed in close spatial proximity to CK8+ tumor-cell areas. Detailed case-level quantification of macrophage marker combinations in the tumor stroma and parenchyma is provided in **Supplementary Figure 4**. To improve readability, the revised main **Figure 6** focuses on representative spatial images and survival analyses.

Kaplan–Meier survival analysis showed that patients with high numbers of IL4I1+ M2-like macrophages in the epithelial/parenchymal tumor region had significantly poorer overall survival than those with low numbers of these cells (*p* = 0.033; **Figure 6B**). A similar but non-significant trend was observed in the stromal area (*p* = 0.082; **Figure 6C**). The total number of M2-like macrophages in the epithelial/parenchymal tumor region also showed a non-significant trend toward poorer survival (p = 0.11; **Figure 6D**).

### IL4I1 expression in M2-like macrophages is associated with altered cytokine and chemokine profiles in the co-culture system

To explore whether IL4I1 expression in M2-like macrophages was associated with changes in the soluble microenvironment, we measured 12 cytokines and chemokines in the supernatants from Huh7 monoculture, Huh7/KO-M2 co-culture, and Huh7/NC-M2 co-culture systems. Detailed cytokine profiling is provided in **Supplementary Figure 5**. Briefly, co-culture with M2-like macrophages altered the secretion of multiple cytokines and chemokines compared with Huh7 monoculture. Compared with Huh7/KO-M2 co-culture, Huh7/NC-M2 co-culture showed higher levels of several inflammatory mediators, including CCL15, CCL17, CCL20, CXCL2, CXCL12, IL-1β, and TNF-α, whereas IL-10 and TGF-β1 were lower. These findings suggest that IL4I1 expression in M2-like macrophages may be associated with changes in the cytokine/chemokine milieu in this *in vitro* co-culture model.

## Discussion

This study characterizes the spatial distribution of IL4I1 expression in macrophages and tumor cells within the HCC TME and provides evidence that IL4I1+ M2-like macrophages are associated with pro-tumorigenic phenotypes *in vitro* and poorer prognosis in patients with HCC. The findings suggest that IL4I1+ M2-like macrophages significantly contribute to the TME in HCC, promoting tumor progression and reducing patient survival. IDO1 and TDO2 are pivotal in initiating the first step of the Kyn metabolic pathway (21). Some cancer cells express high levels of IDO1 and TDO2, utilizing Trp metabolic products to mediate AHR activation, which enhances the malignant phenotype of cancer cells, especially their motility (22, 23). It is now established that the secreted L-amino acid oxidase IL4I1 also produces bioactive metabolites from Trp (15). Given the diversity of Trp catabolizing enzymes in the TME, there is growing interest in developing multi-target inhibition strategies against these enzymes (21, 24, 25). Therefore, understanding the expression and distribution of these metabolic enzymes within TME is crucial for elucidating tumor progression mechanisms and developing targeted therapies.

In this study, through sc-RNA-seq data and mIF results, we found that IL4I1 exhibits a higher expression density in immune cells, particularly macrophages, compared to HCC cells. This observation aligns with previous studies highlighting the central role of TAMs in modulating the TME (16). Conversely, TDO2 is predominantly expressed in liver tumor cells, with minimal expression in immune cells. Within the HCC TME, TDO2 expression is low and sporadically observed in monocytes/macrophages, while IDO2 expression was undetectable in all datasets in TISCH2 (data not shown). In summary, multi-omics data analysis reveals that IL4I1 and TDO2 are the major Trp-catabolizing enzymes in the HCC TME. IL4I1 is primarily expressed in myeloid-derived cells, especially monocytes/macrophages, while TDO2 is mainly expressed in HCC cells. Notably, IL4I1 is highly expressed in the TME compared to adjacent non-tumor tissues, with its high expression correlated with poor prognosis, whereas TDO2 is downregulated in the TME and its low expression is also associated with poor prognosis.

Using the CIBERSORT algorithm, macrophages were estimated to represent a prominent immune cell population within the HCC TME, and elevated IL4I1 expression was associated with macrophage infiltration and poorer patient outcomes. In our THP-1-derived M2-like macrophage/Huh7 co-culture model, IL4I1-expressing M2-like macrophages were associated with increased Huh7 cell proliferation and migration and reduced apoptosis, suggesting a potential macrophage-associated effect on tumor-cell phenotypes *in vitro*. These observations may be explained by at least two non-mutually exclusive mechanisms. First, M2-like macrophages may reshape the soluble microenvironment by secreting cytokines and chemokines that act on neighboring tumor cells and activate tumor-promoting pathways, such as STAT3-related signaling (26). Second, based on previous studies, IL4I1 may generate tryptophan-derived metabolites capable of activating AHR-related signaling, thereby influencing tumor progression and immune regulation (15). However, the present study did not directly quantify IL4I1-derived metabolites or assess AHR activation and downstream targets in HCC cells. Therefore, the IL4I1-AHR axis should be interpreted as a plausible mechanistic hypothesis rather than a pathway proven by the current data.

While transcriptomic data indicated predominant myeloid IL4I1 mRNA expression (**Figures 1**, **2**), direct protein visualization *in situ* using mIF confirmed its presence in both macrophages and some tumor cells (**Supplementary Figures 3A–F**), reflecting the complexities beyond mRNA levels. Building upon these observations, mIF analysis on clinical samples from 92 HCC patients revealed that a higher density of IL4I1+ M2-like macrophages in the tumor parenchyma is correlated with poorer overall survival, suggesting that IL4I1+ M2-like macrophages could serve as a prognostic marker for HCC. Moreover, multivariate Cox regression analysis confirmed that high IL4I1 expression is an independent risk factor for worse prognosis, underscoring the potential clinical significance of targeting IL4I1+ M2-like macrophages in therapeutic strategies.

Besides the observed changes in HCC cell behavior, our cytokine analysis suggests that IL4I1 expression in M2-like macrophages may be associated with remodeling of the soluble cytokine and chemokine milieu in the co-culture system. These changes provide a possible explanation for the pro-tumorigenic phenotypes observed *in vitro*, although their causal contribution requires further validation. The finding that IL4I1 knockout in M2-like macrophages attenuated the secretion of several of these pro-inflammatory mediators (CCL15, CCL17, CCL20, CXCL2, CXCL12, IL-1β, and TNF-α) provides experimental evidence supporting the direct involvement of IL4I1 in shaping the cytokine milieu of the TME. In HCC, these secreted molecules are known to influence tumor cell proliferation, migration, and the recruitment of other immune cells that can promote tumor growth (27–33). This suggests that IL4I1 may exert its pro-tumorigenic effects, at least in part, by modulating the inflammatory secretome of M2-like macrophages, thereby fostering a more permissive environment for HCC progression.

The cytokine findings also suggest that IL4I1 expression in M2-like macrophages may be associated with a mixed or non-canonical TAM-like secretory phenotype rather than a purely classical M2 immunosuppressive program. Although IL-10 and TGF-β1 were lower in the Huh7/NC-M2 co-culture supernatants than in the Huh7/KO-M2 co-culture supernatants, several inflammatory and chemotactic mediators, including CCL15, CCL17, CCL20, CXCL2, CXCL12, IL-1β, and TNF-α, were increased. This pattern indicates that IL4I1-associated macrophage–tumor crosstalk may involve inflammatory and chemotactic remodeling of the soluble microenvironment, rather than simply increasing canonical immunosuppressive cytokines. Moreover, cytokine concentrations in co-culture supernatants represent the net result of production, consumption, and feedback regulation by both macrophages and Huh7 cells. Therefore, the lower IL-10 and TGF-β1 levels should not be interpreted as excluding a pro-tumorigenic role of IL4I1-expressing M2-like macrophages, but rather as evidence that their cytokine phenotype is context-dependent and more heterogeneous than a conventional M2 profile.

Besides the observed changes in HCC cell behavior, our cytokine analysis suggests that IL4I1 expression in M2-like macrophages may be associated with remodeling of the soluble cytokine and chemokine milieu in the co-culture system. Compared with Huh7/KO-M2 co-cultures, Huh7/NC-M2 co-cultures showed higher levels of several inflammatory and chemotactic mediators, including CCL15, CCL17, CCL20, CXCL2, CXCL12, IL-1β, and TNF-α. These molecules have been reported to influence tumor-cell proliferation, migration, immune-cell recruitment, and tumor-promoting inflammation in HCC and other tumor contexts (27–33). Therefore, IL4I1-associated macrophage–tumor crosstalk may contribute, at least in part, to the pro-tumorigenic phenotypes observed *in vitro* by reshaping the inflammatory secretome of the co-culture microenvironment. Notably, IL-10 and TGF-β1 were lower in Huh7/NC-M2 co-culture supernatants than in Huh7/KO-M2 co-culture supernatants, indicating that the IL4I1-associated cytokine pattern should not be interpreted as a purely classical M2 immunosuppressive program. Instead, it may reflect a mixed or non-canonical TAM-like secretory phenotype characterized by inflammatory and chemotactic remodeling rather than simple upregulation of canonical immunosuppressive cytokines (34, 35). Moreover, cytokine concentrations in co-culture supernatants represent the net result of production, consumption, and feedback regulation by both macrophages and Huh7 cells. Thus, the lower IL-10 and TGF-β1 levels do not exclude a pro-tumorigenic role of IL4I1-expressing M2-like macrophages, but rather highlight the context-dependent and heterogeneous nature of their cytokine phenotype. Nevertheless, the causal contribution of these soluble mediators requires further validation through time-course assays, cell-type-specific cytokine profiling, and cytokine-blocking experiments.

Interestingly, our results also showed a significant reduction in the levels of IL-10 and TGF-β1 in both co-culture systems compared to Huh7 monoculture, with the lowest concentrations observed in the Huh7/NC-M2 co-culture. Given that IL-10 and TGF-β1 are generally considered to have immunosuppressive or regulatory functions in the TME, their reduced secretion, particularly in the presence of IL4I1, is noteworthy (36–38). The lower levels of these factors in the NC-M2 condition compared to KO-M2 further imply that IL4I1 expression influences the secretion profile of these regulatory cytokines. While the precise implications of reduced IL-10 and TGF-β1 in this specific context require further investigation, this finding highlights the complex role of IL4I1 in shaping the soluble milieu of the TME beyond simply enhancing pro-inflammatory signals.

An intriguing observation from our bioinformatics analysis was the positive correlation between IL4I1 expression and the infiltration levels of multiple immune cell types, including those often associated with anti-tumor activity such as CD8^+^ T cells (**Figures 4B, C**). This finding might initially appear paradoxical, given the established immunosuppressive functions of IL4I1 via tryptophan catabolism and AHR activation, its association with M2-like macrophages, and our own results demonstrating that high IL4I1 expression and increased IL4I1^+^ M2-like macrophages density are linked to poor prognosis in HCC. However, this apparent contradiction may be reconciled by considering the multifaceted roles of IL4I1-expressing cells within the complex TME. Our cytokine analysis revealed that IL4I1+ M2-like macrophages secrete a broad array of chemokines known to recruit diverse immune populations (**Supplementary Figure 5**). Therefore, the observed positive correlation likely reflects this chemokine-mediated recruitment, leading to increased gross immune cell numbers in IL4I1-high tumors (39). Crucially, this increased infiltration does not necessarily equate to effective anti-tumor immunity; the potent immunosuppressive mechanisms driven by IL4I1 likely impair the function of these recruited cells, contributing to the overall pro-tumorigenic environment and adverse patient outcomes associated with high IL4I1 levels (40). This highlights the complexity of interpreting immune infiltration data and underscores the importance of considering the functional state, rather than just the quantity, of immune cells within the TME.

Our analysis of bulk tumor transcriptomic data provided additional context for the relationship between IL4I1 expression and the broader HCC immune landscape. High IL4I1 expression was associated with signatures of increased immune activity and infiltration, including higher CIBERSORT-estimated proportions of CD8+ T cells and Tregs, as well as elevated chemokine and cytolytic activity scores. However, this immune-enriched phenotype did not translate into improved prognosis, suggesting that increased immune-cell infiltration alone may not reflect effective anti-tumor immunity (41). The apparent discrepancy between the CIBERSORT and mIF findings may be explained by the different nature of these two approaches. CIBERSORT estimates the relative proportions of predefined immune-cell populations from bulk RNA-seq data and does not resolve absolute cell density, spatial localization, or marker co-expression at the single-cell level (42). In contrast, our mIF analysis directly quantified IL4I1+CD68+CD163+ M2-like macrophages within defined tumor regions. Therefore, the mIF-defined IL4I1+ M2-like macrophage population represents a spatially defined macrophage subset rather than the total M2-like macrophage compartment estimated by CIBERSORT. Consistent with this interpretation, our additional TCGA-LIHC marker-gene correlation analysis showed that IL4I1 was positively correlated with several macrophage/TAM-associated markers, including CD68, CSF1R, LST1, CD163, MSR1, MS4A4A, and VSIG4, while its correlation with MRC1 was positive but relatively weak (**Supplementary Figure 6**). The positive correlations of IL4I1 with CD86, IL1B, and CXCL9 further suggest that IL4I1 may be associated with a mixed or heterogeneous macrophage activation state rather than a purely canonical M2 program (34). Together, these findings support the interpretation that IL4I1+ M2-like macrophages represent a specific spatially and phenotypically defined macrophage subset with prognostic relevance, rather than a direct surrogate for the overall CIBERSORT-estimated M2-like macrophage fraction.

These findings support the hypothesis that IL4I1+ M2-like macrophages contribute to a pro-tumorigenic environment. The upregulation of IL4I1+ M2-like macrophages within the TME likely facilitates tumor progression through immunosuppressive mechanisms and close spatial proximity with cancer cells (26). While recent studies have focused on the role of IL4I1 in tumor cells during HCC progression, the contribution of IL4I1-expressing macrophages to this process remains relatively under-researched (43, 44). Given the high expression density of IL4I1 in macrophages, which correlated with poorer patient outcomes. Our study offers new insights for future research in this area. The phenotypic versatility of macrophages within the TME allows them to interact with various cell types, including tumor cells, T cells, endothelial cells (ECs), and fibroblasts, ultimately promoting malignancy by facilitating immune escape and tumor progression (45). A recent study demonstrated that IL4I1^+^PD-L1^+^IDO1^+^ TAMs at the tumor periphery exert immunosuppressive functions by interacting with T cells (16). In contrast, our mIF analysis showed that IL4I1+ M2-like macrophages were located in close spatial proximity to tumor cells within the HCC parenchyma. Together with the co-culture findings, these results suggest that IL4I1-expressing M2-like macrophages may influence tumor-cell phenotypes through paracrine cytokine, chemokine, and/or metabolic crosstalk, thereby contributing to a pro-tumorigenic microenvironment. However, direct cell-cell interaction and activation of the IL4I1-AHR signaling axis were not formally demonstrated in the present study.

The association of IL4I1+ M2-like macrophages with adverse outcomes suggests that this macrophage subset may represent a biologically relevant component of the HCC immune microenvironment and a potential candidate for future therapeutic investigation. However, given the absence of *in vivo* loss- or gain-of-function validation in the present study, IL4I1 should not yet be considered a validated therapeutic target in this context. Future studies are needed to determine whether modulation of IL4I1 activity or its related metabolic pathways can attenuate macrophage-associated pro-tumorigenic effects and improve responses to existing immunotherapies. In particular, integration of IL4I1-focused strategies with approaches targeting heterogeneous TAM populations may be worth exploring, but this possibility requires rigorous preclinical validation (46). More broadly, studying IL4I1+ M2-like macrophages may contribute to a better understanding of macrophage–tumor and macrophage–immune cell crosstalk in HCC and other tumors with immunosuppressive microenvironments (16). These findings therefore support further mechanistic and translational investigation of IL4I1-associated macrophage biology, while emphasizing that therapeutic implications remain preliminary.

Several limitations should be considered when interpreting our findings. First, the functional experiments were performed using a THP-1-derived macrophage/Huh7 co-culture model. Although this system allowed stable CRISPR/Cas9-mediated IL4I1 knockout and controlled comparison between KO-M2 and NC-M2 conditions, THP-1 cells are leukemia-derived and may not fully recapitulate the phenotype, metabolic features, cytokine profile, and functional plasticity of primary human macrophages or bona fide TAMs in HCC tissues. Similarly, the use of Huh7 cells alone cannot capture the biological heterogeneity of HCC. Therefore, the *in vitro* co-culture findings should be interpreted as preliminary functional evidence supporting a potential association between IL4I1 expression in M2-like macrophages and pro-tumorigenic HCC cell phenotypes, rather than definitive proof of tumor-promoting activity across HCC contexts. Future studies using primary human monocyte-derived macrophages, macrophages isolated from HCC patients, and additional HCC cell lines are needed to validate the clinical relevance and generalizability of these findings. Second, the present study lacks *in vivo* validation. Co-culture assays cannot fully reproduce the spatial, metabolic, vascular, and immune complexity of the HCC microenvironment. Thus, whether IL4I1+ M2-like macrophages contribute to tumor growth, metastasis, immune remodeling, or therapeutic response *in vivo* remains to be determined. Future studies using orthotopic or syngeneic HCC models, macrophage-specific IL4I1 loss- or gain-of-function approaches, or adoptive transfer of IL4I1-modified macrophages will be required to clarify the physiological relevance of this macrophage subset. Third, the IL4I1-AHR signaling axis was not directly validated in this study. Although previous studies have shown that IL4I1 can generate tryptophan-derived metabolites capable of activating AHR, we did not quantify kynurenine, kynurenic acid, indole-3-aldehyde, or other IL4I1-related metabolites in the co-culture supernatants. In addition, AHR activation in HCC cells, including nuclear translocation and downstream targets such as CYP1A1, CYP1B1, and AHRR, was not assessed, and rescue or inhibition experiments using AHR antagonists or IL4I1-related metabolites were not performed. Therefore, the IL4I1-AHR axis should be interpreted as a plausible mechanistic hypothesis rather than a pathway proven by the current study. Further studies integrating metabolite profiling, AHR pathway assays, cell-type-specific transcriptomic analysis after co-culture, and functional rescue or blockade experiments are needed to define the molecular mechanisms by which IL4I1-expressing M2-like macrophages may influence cytokine remodeling and tumor-cell behavior. In addition, the therapeutic relevance of IL4I1 inhibition requires rigorous preclinical validation before translation into clinical applications can be considered.

## Conclusion

In the current study, our findings suggest that IL4I1+ M2-like macrophages are associated with adverse patient outcomes and may contribute to a pro-tumorigenic microenvironment in HCC. The combination of public single-cell analyses, *in vitro* co-culture assays, cytokine profiling, and mIF-based spatial analysis supports the potential prognostic relevance of this macrophage subset. However, the functional evidence was derived from a THP-1-derived macrophage/Huh7 co-culture model and lacks validation using primary macrophages, additional HCC cell lines, and *in vivo* models. Moreover, the IL4I1-AHR signaling axis was not directly examined, as IL4I1-derived tryptophan metabolites, AHR activation, and downstream target genes were not assessed. Therefore, IL4I1+ M2-like macrophages should be regarded as a prognostically relevant and biologically plausible contributor to HCC progression, while further mechanistic and *in vivo* studies are required before IL4I1 or its metabolic pathway can be considered a validated therapeutic target in HCC.

## Data Availability

Publicly available datasets were analyzed in this study. These data can be found in the GEO database under accession numbers GSE140228, GSE146409, and GSE166635, and in the TCGA-LIHC cohort accessed through TIMER, UALCAN, and KM plotter. Original experimental data generated during this study are available from the corresponding author upon reasonable request.

## Glossary

AHR: Aryl Hydrocarbon Receptor
ANOVA: Analysis of Variance
CAFs: Cancer-Associated Fibroblasts
CCL2: Chemokine (C-C motif) ligand 2
CCL15: Chemokine (C-C motif) ligand 15
CCL17: Chemokine (C-C motif) ligand 17
CCL20: Chemokine (C-C motif) ligand 20
CK8: Cytokeratin 8
CRISPR/Cas9: Clustered Regularly Interspaced Short Palindromic Repeats/Cas9
CTLA-4: Cytotoxic T-lymphocyte-associated protein 4
CXCL2: Chemokine (C-X-C motif) ligand 2
CXCL12: Chemokine (C-X-C motif) ligand 12
CYT: Cytolytic activity score
DAPI: 4’,6-diamidino-2-phenylindole
ECs: Endothelial Cells
ELISA: Enzyme-Linked Immunosorbent Assay
GAPDH: Glyceraldehyde-3-phosphate dehydrogenase
GEO: Gene Expression Omnibus
HCC: Hepatocellular Carcinoma
HEK293T: Human embryonic kidney 293T cell line
HR: Hazard Ratio
I3A: Indole-3-aldehyde
ICIs: Immune Checkpoint Inhibitors
IDO1: Indoleamine 2,3-dioxygenase 1
IL-1β: Interleukin-1 beta
IL-4: Interleukin-4
IL-6: Interleukin-6
IL-8: Interleukin-8
IL-10: Interleukin-10
IL4I1: Interleukin-4-induced gene 1
Kyn: Kynurenine
KynA: Kynurenic Acid
MDSCs: Myeloid-Derived Suppressive Cells
MeTIL: Melanoma-associated T cell Infiltration score
mIF: Multiplex Immunofluorescence
OS: Overall Survival
PBMCs: Peripheral Blood Mononuclear Cells
PD-1: Programmed cell death-1
PFS: Progression-Free Survival
PMA: Phorbol 12-myristate 13-acetate
PPI: Protein-Protein Interaction
sc-RNA-seq: Single-cell RNA sequencing
SD: Standard Deviation
sgRNA: Single guide RNA
ST: Spatial Transcriptomics
STAT3: Signal transducer and activator of transcription 3
TAMs: Tumor-Associated Macrophages
TBST: Tris-Buffered Saline with Tween-20
TDO2: Tryptophan 2,3-dioxygenase
TGF-β1: Transforming growth factor beta 1
TIMER: Tumor Immune Estimation Resource
TISCH2: Tumor Immune Single-cell Hub 2
TKIs: Tyrosine Kinase Inhibitors
TMA: Tissue Microarrays
TME: Tumor Microenvironment
TNF-α: Tumor necrosis factor alpha
Tregs: Regulatory T cells
VEGFR2: Vascular endothelial growth factor receptor 2
