# Supplementary material for: Association of IL4I1+ M2-like macrophages in tumor microenvironment with poor prognosis in hepatocellular carcinoma: insights from bioinformatics and experimental validation
Source: Front Immunol. 2026 Jul 9;17:1872272. doi: 10.3389/fimmu.2026.1872272 (PMC13391858; doi:10.3389/fimmu.2026.1872272)
Supplement: Supplementary file 1 [file SupplementaryFile1.pdf]

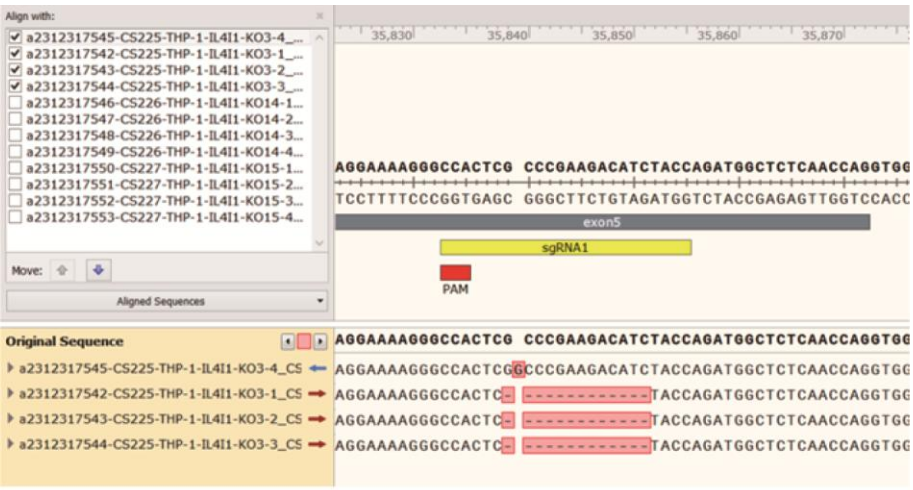

Supplementary Figure 1. Validation of IL4I1 gene knockout in THP1 cells (KO#3 clone). Sequence analysis of the genomic DNA from the established THP1 single-cell clone (KO#3) demonstrating successful knockout of the IL4I1 gene (Gene ID: 259307). The analysis reveals a 13 bp deletion and a 1 bp (G) insertion in the DNA double strands of the KO#3 clone. These non-multiple-of-three indels result in a frameshift mutation, confirming the successful disruption of the IL4I1 coding sequence and gene knockout. The figure shows an alignment of the edited sequence from the KO#3 clone compared to the original sequence, highlighting the specific indel events.

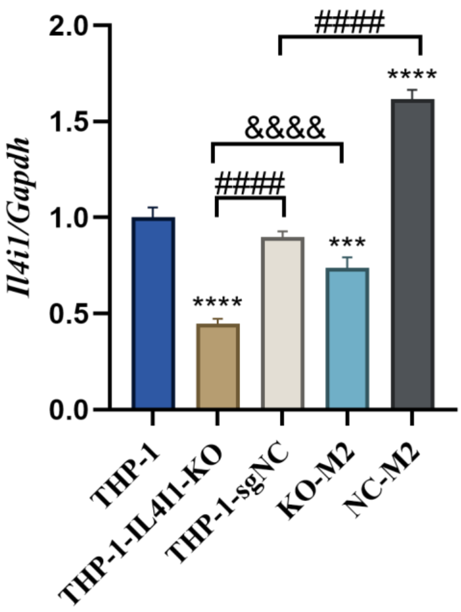

Supplementary Figure 2. Relative IL4I1 gene expression in various THP-1 cell lines and polarization states. Relative mRNA expression levels of IL4I1, normalized to the housekeeping gene GAPDH, were determined by RT-qPCR in parental THP-1 cells, an IL4I1 knockout clone (THP-1-IL4I1-KO), a negative control sgRNA clone (THP-1-sgNC), and these cells after M2 polarization (KO-M2 and NC-M2). Data are presented as mean ± SD. Statistical significance is indicated as follows: * denotes comparison with THP-1 group: *p<0.05, **p<0.01, ***p<0.001, ****p<0.0001; # denotes comparison with THP-1-sgNC group: #p<0.05, ##p<0.01, ###p<0.001, ####p<0.0001; & denotes comparison with THP-1-IL4I1-KO group: &p<0.05, &&p<0.01, &&&p<0.001, &&&&p<0.0001.

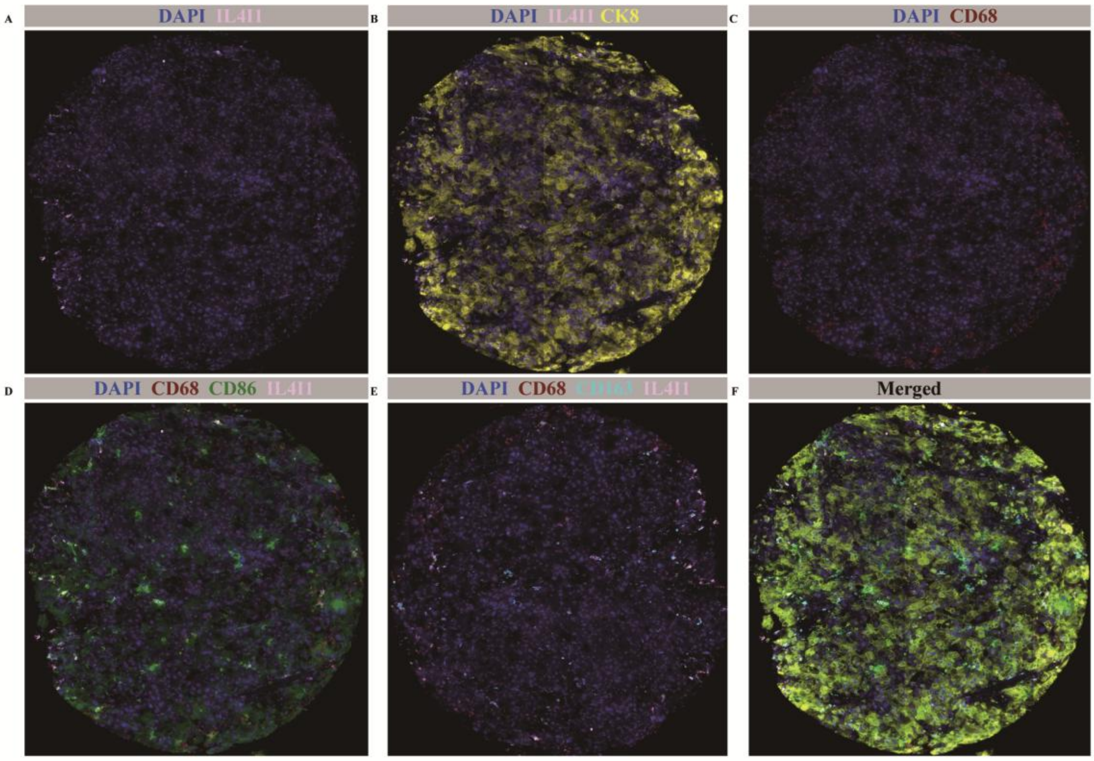

Supplementary Figure 3. Polarization of THP-1 macrophages. (A) IL4I1 (pink) and DAPI (blue) staining show the localization of IL4I1+ cells in the tissue section. (B) Co-staining of IL4I1 (pink), CK8 (yellow), and DAPI (blue) demonstrates the distribution of IL4I1+ cells among CK8+ tumor cells. (C) CD68 (red) and DAPI (blue) staining indicate the presence of macrophages in the tissue. (D) Multicolor staining of IL4I1 (pink), CD68 (red), CD86 (green), and DAPI (blue) highlights the presence of M1 macrophages (CD68+CD86+) and IL4I1 expression. **(**E**)** Staining of IL4I1 (pink), CD68 (red), CD163 (cyan), and DAPI (blue) reveals the presence of M2 macrophages (CD68+CD163+) and IL4I1 expression. (F) Merged image of all markers showing the spatial relationship between IL4I1, CK8, CD68, CD86, CD163, and DAPI in the HCC tissue section.

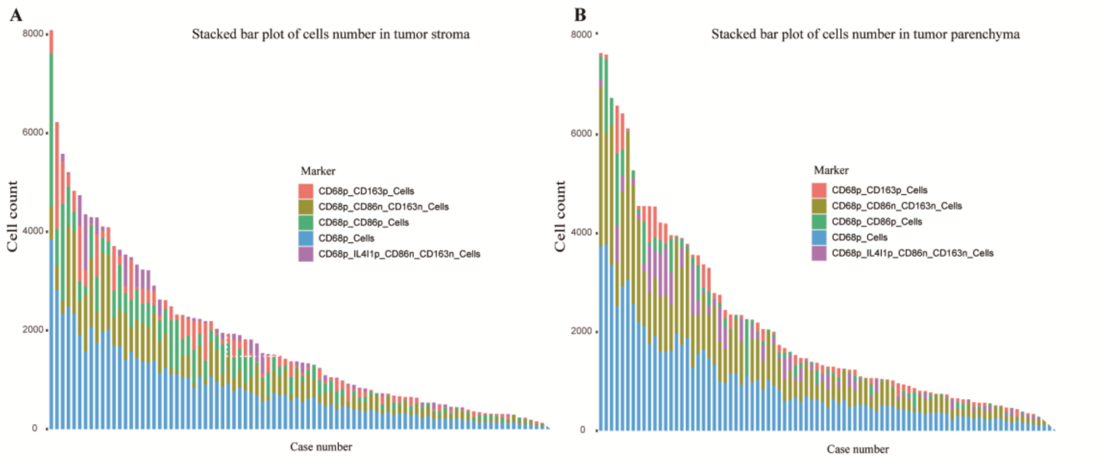

Supplementary Figure 4. Case-level quantification of macrophage marker combinations in HCC tissues. (A-B) Stacked bar plots showing the number of cells with different marker combinations in the tumor stroma and tumor parenchyma for each individual case. These plots provide detailed case-level quantification supporting the mIF analysis summarized in Figure 6.

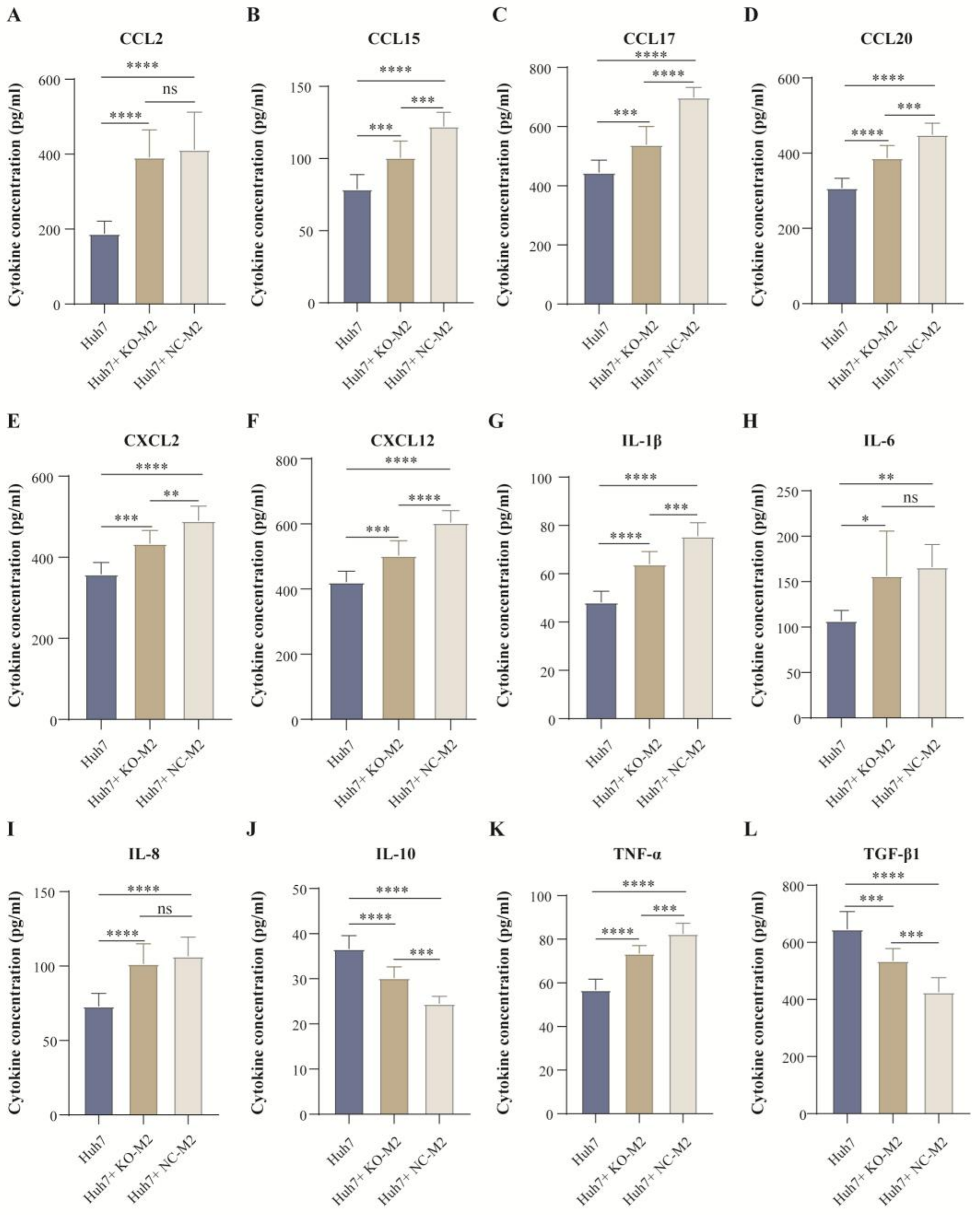

Supplementary Figure 5. Cytokine and chemokine concentrations in supernatants from Huh7 monoculture and co-culture systems. The concentrations of (A) CCL2, (B) CCL15, (C) CCL17, (D) CCL20, (E) CXCL2, (F) CXCL12, (G) IL-1β, (H) IL-6, (I) IL-8, (J) IL-10, (K) TNF-α, and (L) TGF-β1 were measured by ELISA in culture supernatants collected after 72 hours of culture from Huh7 monoculture, Huh7/KO-M2 co-culture (Huh7 cells co-cultured with IL4I1 knockout M2 macrophages), and Huh7/NC-M2 co-culture (Huh7 cells co-cultured with negative control M2 macrophages). Data are presented as mean ± SD. Statistical significance was determined by one-way ANOVA with Tukey's post-hoc test. **p*<0.05, ***p*<0.01, ****p*<0.001, *****p*<0.0001, ns = not significant.

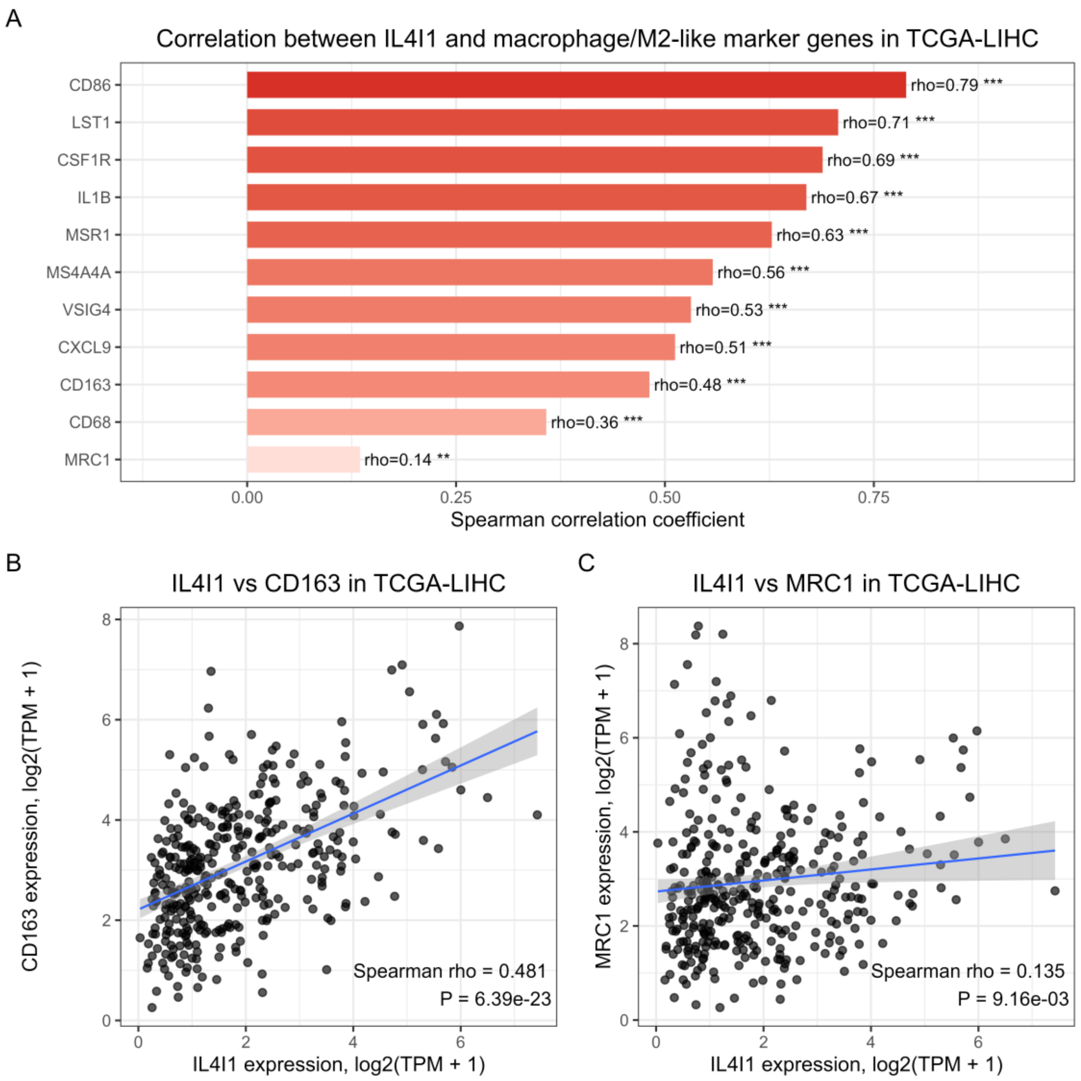

Supplementary Figure 6. Correlations between IL4I1 and macrophage/M2-like marker genes in TCGA-LIHC.

(A) Spearman correlation coefficients between IL4I1 and representative macrophage, M2-like, or TAM-associated marker genes in TCGA-LIHC primary tumor samples. (B) Scatter plot showing the correlation between IL4I1 and CD163 expression. (C) Scatter plot showing the correlation between IL4I1 and MRC1 expression. Gene expression values are shown as log2(TPM + 1). Spearman's rho and p values are indicated.***, *p*-adj< 0.001; **, *p*-adj< 0.01.
